# Patient-Reported Health Outcomes After Treatment of COVID-19 with Nebulized and/or Intravenous Neutral Electrolyzed Saline Combined with Usual Medical Care Versus Usual Medical care alone: A Randomized, Open-Label, Controlled Trial.

**DOI:** 10.21203/rs.3.rs-68403/v1

**Published:** 2020-09-10

**Authors:** Ivan Delgado-Enciso, Juan Paz-Garcia, Carlos E Barajas-Saucedo, Karen A Mokay-Ramírez, Carmen Meza-Robles, Rodrigo Lopez-Flores, Marina Delgado-Machuca, Efren Murillo-Zamora, Jose A. Toscano-Velazquez, Josuel Delgado-Enciso, Valery Melnikov, Mireya Walle-Guillen, Hector R. Galvan-Salazar, Osiris G. Delgado-Enciso, Ariana Cabrera-Licona, José Guzman-Esquivel, Daniel A. Montes-Galindo, Alejandra E. Hemandez-Rangel, Patricia Montes-Diaz, Iram P Rodriguez-Sanchez, Margarita L Martinez-Fierro, Idalia Garza-Veloz, Daniel Tiburcio-Jimenez, Sergio A Zaizar-Fregoso, Mario Ramirez-Flores, Gustavo Gaytan-Sandoval, Carlos R. Martinez-Perez, Francisco Espinoza-Gómez, Fabián Rojas-Larios, Michael J. Hirsch-Meillon, Enrique Barrios-Navarro, Vladimir Oviedo-Rodriguez, Luz M. Baltazar Rodriguez, Brenda A. Paz-Michel

**Affiliations:** Universidad de Colima Facultad de Medicina; Centro Hospitalario Unión, Villa de alvarez, colima; Universidad de Colima Facultad de Medicina; Universidad de Colima Faculty of Medicine: Universidad de Colima Facultad de Medicina; Departament of research Cancerology State Institute of Colima; Departament of research Cancerology State Institute of Colima; Departament of research Cancerology State Institute of Colima; Departament of research General Hospital of Zone No. 1 IMSS, Villa de alvarez, Colima; Universidad de Colima Faculty of Medicine: Universidad de Colima Facultad de Medicina; Departament of research Foundation for Cancer Ethics, education and Research of the Cancerology State Institute; Universidad de Colima Facultad de Medicina; Universidad de Colima Facultad de Medicina; Departament of research Cancerology State Institute of Colima; Departament of research foundation for Cancer Ethics, Education and Research of the Cancerology State Institute; Departament of research, Esteripharma.; Departament of research, General Hospital of zone no. 1 IMSS, Villa de Alvarez, Colima; Institute of Cancerology: Instituto de Cancerologia; Universidad de Colima Facultad de Medicina; Private medical office Xochitepec; Departament of research Hospital of zone no.1 imss Villa de alvarez, Colima; Molecular medicine laboratory: Academic unit of human medicine and health sciences; Molecular medicine laboratory, Academic unit of Human medicine and health sciences; Universidad de Colima Faculty of Medicine: Universidad de Colima Facultad de Medicina; Universidad de Colima Faculty of Medicine: Universidad de Colima Facultad de Medicina; Universidad de Colima Faculty of Medicine: Universidad de Colima Facultad de Medicina; Departament of research Cancerology State Institute of Colima; Departament of research Cancerology State Institute of Ciolima; Universidad de Colima Facultad de Medicina; Universidad de Colima Facultad de Medicina; Departament of research Cancerology State Institute of Colima; Universidad de Colima Facultad de Medicina; Departament of research Cancerology State Institute of Colima; Universidad de Colima Faculty of Medicine: Universidad de Colima Facultad de Medicina; Autonomous University of Nueo León

**Keywords:** COVID-19, Neutral Electrolyzed Saline, SARS-CoV-2, reactive oxygen species, reactive chlorine species, immune system, inflammation, treatment

## Abstract

**Background:**

Coronavirus disease (COVID-19) is currently the main public health problem worldwide. The administration of neutral electrolyzed saline, a solution that contains reactive species of chlorine and oxygen (ROS), may be an effective therapeutic alternative due to its immunomodulating characteristics, in systemic inflammation control, as well as in immune response improvement, promoting control of the viral infection. The present study evaluated the efficacy of treatment with intravenous and/or nebulized neutral electrolyzed saline combined with usual medical care *versus* usual medical care alone, in ambulatory patients with COVID-19.

**Methods:**

A prospective, 2-arm, parallel group, randomized, open-label, phase I-II clinical trial included 39 patients in the control group (usual medical care alone) and 45 patients in the experimental group (usual medical care + intravenous and/or nebulized electrolyzed saline, with dose escalation). Two aspects were evaluated during the twenty-day follow-up: i) the number of patients with disease progression (hospitalization or death); and ii) the Patient Acceptable Symptom State (PASS), a single-question outcome that determines patient well-being thresholds for pain and function. Biochemical and hematologic parameters, as well as adverse effects, were evaluated in the experimental group.

**Results:**

The experimental treatment decreased the risk for hospitalization by 92% (adjusted RR=0.08, 95% CI: 0.01–0.50, P=0.007), with a 43-fold increase in the probability of achieving an acceptable symptom state on day 5 (adjusted RR= 42.96, 95% CI: 9.22–200.0, P<0.001). Intravenous + nebulized administration was better than nebulized administration alone, but nebulized administration was better than usual medical care alone. Clinical improvement correlated with a decrease in C-reactive protein, and aberrant monocytes and an increase of lymphocytes, and platelets. Cortisol and testosterone levels were also evaluated, observing a decrease in cortisol levels and an increment of testosterone-cortisol ratio, on days 2 and 4.

**Conclusions:**

The experimental treatment produced no serious adverse effects. In conclusion, intravenous and/or nebulized neutral electrolyzed saline importantly reduced the symptomatology and risk of progression (hospitalization and death), in ambulatory patients with COVID-19.

**Trial registration:**

Cuban Public Registry of Clinical Trials (RPCEC) Database RPCEC00000309. Registered: 05. May 2020. https://rpcec.sld.cu/en/trials/RPCEC00000309-En

## Background

Coronavirus disease (COVID-19), caused by the severe acute respiratory syndrome coronavirus 2 (SARS-CoV-2), is currently the main public health problem worldwide [1][2]. Previous studies reported that the most common initial symptoms are systemic, upper respiratory and cough. Lower respiratory and gastrointestinal symptoms are less frequent and generally appear at the late stage of the disease [3]. The symptoms, if present, of longest duration are cough, loss of sense of smell or taste, sinus congestion, shortness of breath upon exertion, body aches, and headache [3]. A study about the time that COVID-19 patients required to achieve a usual state of health reported that sixty-five percent have returned to their usual state of health 7 days from the date of diagnosed, whereas 35% of patients had not returned to their usual state of health, 12–14 days after receiving a positive test result [4]. Although most infections are self-limited, an estimated 15% of infected adults develop severe pneumonia that requires treatment with supplemental oxygen and hospitalization [5]. Nevertheless, the number of infected patients identified as having severe infection and requiring hospitalization varies among regions and countries, whether due to inherent conditions in the population [6] or to the strategy used in identifying individuals that are positive to the virus [7]. In Mexico, 40.3% of confirmed cases are estimated to require hospitalization [8].

There are numerous experimental proposals for treating COVID-19. Initially, chloroquine showed promise as being a useful treatment, but its lack of efficacy has since been demonstrated [9]. Despite the numerous drugs that are currently recommended, such as nonsteroidal anti-inflammatory drugs (NSAIDs), corticoids, antivirals, antibiotics, and proinflammatory cytokine (interleukin) modulators, no specific drug therapy has yet been proven to be effective against SARS-CoV-2 [10]. Treatment is symptomatic, and oxygen therapy is the first step in addressing respiratory impairment [1]. Noninvasive and invasive mechanical ventilation may be necessary in cases of respiratory failure that is refractory to oxygen therapy [1].

COVID-19 symptomatology and manifestations depend on the degree of immune dysregulation caused by the virus, characterized by systemic inflammation and remote organ injury [11].Viral infection is capable of producing an excessive immune reaction in the host. In severe cases, a reaction known as a ‘cytokine storm’ occurs [1]. A rapid and robust type I IFN-orchestrated response can lead to virus clearance, given that antiviral lymphocytes, such as natural killer (NK) cells, are activated and expanded. Conversely, late activation of innate immunity is usually associated with severe pathology that can lead to pneumonia, acute respiratory distress syndrome (ARDS), septic shock, multi-organ failure, and eventually, death [12]. Different immune system alterations come together to produce severe disease. A key factor in the cytokine storm in COVID-19 is the elevation of monocytes, producing IL-6, a circulating innate immune cell [13], combined with lymphocyte reduction that limits the systemic antiviral response [14][15]. Inefficient SARS-CoV-2 clearance by alveolar macrophages can promote excessive viral replication, leading to severe pathology that is accompanied by increased viral shedding, and in turn, viral transmissibility [12]. We postulate that the administration of intravenous and/or nebulized electrolyzed saline can aid in modulating the body’s immune response to SARS-CoV-2, reducing symptomatology and preventing disease progression.

Electrolyzed saline is produced from a saline solution of sodium chloride, activated by a controlled process of electrolysis, producing reactive species of chlorine and oxygen (ROS). Significant examples of said ROS are oxidant chlorine species, such as hypochlorous acid (HOCI), and oxidant oxygen species, such as hydrogen peroxide (H_2_O_2_). Molecular hydrogen (H_2_) is also produced (Pat. No. MX330845B). ROS are normally produced in the organism and have different physiologic functions [16]. Their most well-known activity is control of bacteria, parasites, and viruses, through the activity of innate immune response cells, macrophages, and neutrophils that release ROS to structurally damage the invading pathogens, thus protecting the host from infection [17].

A series of recent studies have shown that, in addition to the primordial innate immune response, ROS are secondary messengers in processes of exacerbated inflammation control and tissue repair in a process known as redox signaling. Redox signaling is ROS-dependent and the immune response varies, according to ROS concentrations and exposure time [17–21]. Different studies have shown that they can activate and repair phenotypes, such as M2 macrophages and regulatory T cells, acting as potentiators of the humoral immune response [22][23]. ROS have been shown to mediate the communication between the different cells of the immune system, such as polymorphonuclear cells, neutrophils, macrophages, antigen-presenting cells, B cell, and T cells [22][23][24]. Specifically, hypochlorous acid can act as a coadjuvant and adaptive immune response stimulator by modifying antigen proteins, increasing their recognition, processing, and presentation by antigen-presenting dendritic cells [25]. In addition, ROS play an important role in later stages of B cell activation by promoting the sustained signaling of B cell antigen receptors, thus favoring antibody production [26]. Numerous studies have also shown that H_2_ has beneficial effects in diverse animal models and human disease [27]. Its oral administration in an animal model limited the increase of IL-6 and alpha-tumor necrosis factor, producing a potent antioxidant and anti-inflammatory effect [28].

Therefore, the present study was designed to randomly select patients with COVID-19 receiving usual medical care, with the objective of comparing the safety and efficacy of two treatments: the usual medical care combined with electrolyzed saline (administered intravenously and/or through nebulizations, with dose escalation) and the usual medical care alone (control).

## Methods / Design

### Study design

A prospective, randomized, single-blind, 2-arm, parallel group, open-label, dose-scalation, phase I-II clinical trial with allocation ratio 1:1, was conducted between May and June 2020, and carried out according to the “CONSORT statement” guidelines for randomized controlled trials. The study was approved by the ethics committee of the School of Medicine of the *Universidad de Colima*, México (April 8,2020), and written statements of informed consent were obtained from all the participants. The trial was performed in accordance with the principles of the Declaration of Helsinki and the International Conference on Harmonization–Good Clinical Practice guidelines. The present clinical trial was registered as TX-COVID19: RPCEC00000309 in the Cuban Public Registry of Clinical Trials (RPCEC) database (May 5, 2020).

### Study subjects

The inclusion criteria were men and nonpregnant women ≥18 years of age, presenting with COVID-19 and a positive diagnosis of SARS-CoV-2 by RT-PCR, that had a medical consultation due to their illness and were indicated for at-home ambulatory treatment. The women agreed to utilize effective contraceptive measures during the study period and for at least 15 days after the final drug administration of the analysis. Exclusion criteria were pregnant or breastfeeding women and patients presenting with any of the following conditions, prior to the diagnosis of COVID-19: cancer, ischemic heart disease, chronic decompensated systemic disease, creatinine 1.25 times higher than the normal value or creatinine clearance below 50 milliliters/minute (Cockcroft-Gault method), blood hemoglobin below 10 g/dl, drug addiction (illegal drugs), or known liver disease with a doubling of liver function test values (aspartate aminotransferase (AST), alanine aminotransferase (ALT), alkaline phosphatase, or bilirubin). Additionally, the following elimination criteria were used: patients that voluntarily decided to abandon the study, patients that, at some point of the study presented with severe toxicity (grade 3 or higher, according to the common terminology criteria for adverse events, CTCAE v5.0, U.S. Department of Health and Human Services), that was attributable to the administration of the experimental drug.

The physicians participating in the project identified candidates from primary and secondary healthcare centers (public or private) in the Mexican states of Colima and Morelos. The physicians asked the patients for their permission, once they were at home, for the researchers to call them by telephone, requesting their participation in the study. Before said phone call, the candidates were randomly allocated to the experimental group (electrolyzed saline + usual medical care) or the control group (usual medical care alone). Randomization was performed using computer-generated random allocation cards. In that manner, the patients were directly asked to participate in one of the non-blinded groups. Blinded patient inclusion (experimental *vs* placebo) had initially been planned but none of the patients accepted the potential administration of a placebo. As a result, the protocol was modified, and the patients were randomized before making the phone calls. The inclusion process was conducted by researchers that did not participate in the evaluation of the results. Before entering the study, all the patients were receiving usual treatment, under the care of their family physician or specialist. When asked to participate in the study, the patients selected for the electrolyzed saline group were told they would receive an experimental treatment in addition to their usual medical care, as well as have sign and symptom follow-up and undergo certain laboratory tests. The patients receiving usual medical care (control group) were asked to participate in the study, through sign and symptom follow-up conducted by telephone. All the patients were advised that they would continue to be under the supervision of their regular physician or healthcare institution and that the research team, would in no way, modify or limit any intervention that their physician, or they themselves, considered pertinent, such as going to the emergency service if there were alarm symptoms.

### Electrolyzed Saline Administration

The experimental treatment consisted of an aqueous saline solution of sodium chloride, activated by a controlled process of electrolysis (Pat. No. MX330845B), and thus was recognized as activated saline, electrolyzed saline or electrolyzed water. It had a neutral pH (6.0–7.5) and its active ingredient was 0.002% of active species of chlorine and oxygen. The Good Manufacturing Practices (GMP) for intravenous electrolyzed saline (HOMEOSTECH®) also met the required processes for sterile injectable products. As an IV electrolyzed saline, its formulation was 17.12 mEq/L of sodium chloride and 0.38 mM of active species of chlorine and oxygen. The vials utilized were 5 ml ampules, and the name and composition were indelibly printed on each one. The electrolyzed saline was provided by *Esteripharma S.A. de C.V.*, (México City, Mexico).

When the randomized patient corresponded to the electrolyzed saline group, he or she was included in a “rolling six” dose escalation design, as has previously been reported [29]. Dose level 1 consisted of nebulizations. The nebulizations were indicated 4 times a day for 10 days. They were performed by placing 5 ml of electrolyzed saline in the nebulizer chamber and continuing the nebulization until the content was used up (10–15 min). The nebulizations were carried out following the recommendations of the American College of Allergy, Asthma and Immunology [30], the British Lung Foundation [31], the Asthma Society of Ireland [32], and the British Thoracic Society [33]. They were conducted with no assistants, in a well-ventilated room or an outside area, such as a patio or garage, where the air did not recirculate into the house, and with adequate cleansing of the device. The nebulizations were included in the subsequent dose levels (combined intravenous and nebulized administration).

The intravenous dose began with a dose within a safe range, previously established in a phase I clinical trial conducted at the *Institute Estatal de Cancerología de Colima* for the treatment of chikungunya (data not published) by the present research group. The initial applications were 15 ml (dose level 2) once a day for 7 days, with successive increases, before reaching dose-limiting toxicity (DLT), or until a dose was found that prevented disease progression. The first two increases were approximately 40%: 20 ml/day (dose level 3) and 30 ml/day (dose level 4). Upon reaching the dose of 30 ml per day, efficacious symptomatology resolution was observed in the majority of ambulatory patients, and so was maintained in a high number of patients.

If a patient did not respond favorably to those regimens, an accelerated titration dose-escalation design was established, but only in patients whose clinical evolution suggested a high risk for disease progression. Those were, a) patients that after a period of three days of continuous improvement with the administration of the dose level 4 scheme, began to have a clinically relevant increase (30%) in symptom severity (myalgia, arthralgia, dyspnea), with the addition of diarrhea and/or intense nausea or vomiting, that lasted for at least 24–48 h, or b) patients with severe symptomatology sustained for more than 7 days, before beginning the study treatment. The scaling (defined as dose level 5) involved 100% increases of the dose (applications every 12 h, rather than every 24 h) in single-patient cohorts until there was dose-limiting toxicity or an effective dose for preventing disease progression was achieved. That design suggested the possibility of intra-patient dose escalation, for patients included in the protocol receiving the dose of 30 ml (dose level 4). The dose level 5 scheme was begun immediately in all new patients that were candidates for it. The first patient treated with that regimen had begun to have a worsening of symptoms, despite having received 5 days of treatment with 30 ml (dose level 4). The 30 ml dose was maintained but administered every 12 h for three days (dose level 5.1). Said patient was hospitalized, resulting in later patients that met the dose level 5 criterion receiving 30 ml every 12 h for 6 days (dose level 5.2). If those patients were not in satisfactory condition on the sixth day of treatment, 3 more days of application of 40 ml of electrolyzed saline, every 12 h (dose level 5.3, 9 days of application, every 12 h) were added. To finalize all level five doses (dose levels 5.1–5.3), 2 more days of 30 ml per day were added.

The highest dose (dose level 5.3) was tested on a single patient, who after 2 days with the dose level 4 scheme (30 ml per day), had the dose increased to 30 ml every 12 h for 6 days, followed by 40 ml every 12 h for 3 days, finalizing treatment with 30 ml every 24 h for 2 more days, and achieving recovery.

A patient on the dose level 2 scheme, after 3 days of treatment, was hospitalized and intubated the same day of his admission, with both the patient and relatives requesting that the experimental treatment be continued. Authorized by the ethics committee, the patient received 30 ml every 12 h for the 10 days he was intubated, 20 ml every 12 h for 6 more days of hospitalization, but no intubation, finalizing treatment at home with 15 ml per day for 6 days.

One-third of the volume of the electrolyzed saline was diluted with physiologic saline solution (0.9% of NaCl), right before its application. The solution was administered intravenously in bolus (passing it in 1 – 2 minutes), utilizing a 25G, 0.5 × 15 mm venipuncture set, connected to a syringe. Two patients with applications twice a day for 6 days were cannulated to maintain venous patency. In 4 patients with applications once a day, a heparinized peripheral venous catheter was placed for its intermittent use.

In the final month of the protocol and after approval of the modification by the Ethics Committee, new therapeutic administration pathways of electrolyzed saline were added to rapidly evaluate their effects on certain particular signs and symptoms. If there was nausea, vomiting and/or diarrhea, 30 ml of oral electrolyzed saline was added, 4 times a day, for as long as symptoms lasted, plus 2 more days after symptoms disappeared. In patients with oropharyngeal ulcerations (causing intense throat pain), the indication was to gargle with 10 ml of electrolyzed saline, 6 times a day, and swallow the solution after gargling with it. This was done for the number of days necessary for the pain to decrease to 4 or less on the visual analog scale (VAS). Said scale values are from 0 to 10, in which 0 is no pain and 10 is the maximum pain tolerable. The oral pathway was indicated in 5 patients and gargling was indicated in 6. The addition of new treatments during the course of a protocol to rapidly evaluate new therapies without compromising the original trial outcomes has been considered adequate in previous scientific reviews [34].

### Usual medical care

The patients receiving only usual medical care continued with the usual treatment prescribed by their family physician or specialist. It consisted of the administration of paracetamol, nonsteroidal anti-inflammatory drugs (NSAIDs), steroids, azithromycin, chloroquine, ivermectin, and/or antiviral drugs, etc., with the indication to return to the emergency service if there was respiratory difficulty or worsening of symptomatology. The researchers did not intervene in drug prescription or lifestyle indications.

### Outcome Measures and Follow-up

There were 3 co-primary endpoints. The first was the number of patients with disease progression, defined as hospitalization or death. The second primary end point was the Patient Acceptable Symptom State (PASS), defined as the value of symptoms the patient considered to be well-being thresholds of pain and function. Our study incorporated the most widely used anchoring question to identify PASS cut-off points, which was: “Taking into account all your daily activities, do you consider your current state satisfactory in relation to pain level and functional impairment?” The response options were “Yes” or “No” [35][36](34)[37]. Treatment success was defined as no disease progression, or a PASS answered in the affirmative on days 3, 5, or 7 of follow-up. The third endpoint was the change from the baseline in the patient overall self-assessment, which was determined by the response to the question: Considering all the ways in which illness and health conditions may affect you at this time, please indicate below how you are doing? The response options were measured on the 0–10 VAS, from ‘very well’ (score of 0) to ‘very poorly’ (score of 10). That question is validated in the Routine Assessment of Patient Index Data 3 (RAPID3), which is a pooled index of the three patient-reported Core Data Set measures of the American College of Rheumatology, and it has previously been used to determine the activity of autoimmune diseases, degenerative diseases, such as osteoarthritis (18), and infectious diseases with a strong component of general malaise, such as chikungunya fever (37). That end point is similar to the symptom severity score (self-assessed using a 10-point VAS) recently used in a clinical trial that evaluated the efficacy of hydroxychloroquine in non-hospitalized patients with COVID-19 [38]. The secondary endpoints were changes from the baseline in different types of body pain (arthralgia, myalgia, headache, and sore throat), or more exactly, the difference between the values at enrollment and on days 3, 5, and 7 of follow-up. Pain was measured on the 0–10 VAS (27). Intensity of pain was recorded, from ‘no pain’ (score of 0) to ‘worst imaginable pain’ (score of 10). The VAS was selected because it is the validated scale that best evaluates pain in diseases (28,29), at present, and because it has also been used as a primary endpoint in other clinical trials (30). Patients completed the previously validated fatigue VAS (0–10 scale) [37], which states: “How much of a problem has unusual fatigue or tiredness been for you today” and was anchored from 0 (fatigue is not a problem) to 10 (fatigue is a major problem). Daily coughing episodes were reported by the patient on a numerical scale from 0 to 20. If there were more than 20 episodes, they were registered as 20. Dyspnea was determined once a day through the Borg scale, from 0 to 10, in which 0 is no dyspnea and 10 is extremely severe dyspnea [37]. Nausea, vomiting, diarrhea, dizziness, conjunctivitis, rhinorrhea, exanthema, skin rash, and loss of sense of smell or taste were catalogued as present or absent for each day of follow-up. Adverse events were monitored by the researchers through anamnesis and abnormal routine laboratory test results. Follow-up was carried out for at least 20 days or until patient outcome (cure or death). Daily follow-up was suspended in the hospitalized patients, and from the day of hospital admission, their registers were considered lost data and were not considered in the analysis from that day forward, with the exception of the PASS, in which its subsequent registers were reported as a negative acceptable symptom state. Nevertheless, the general aspects of those patients were registered, such as hospitalization and outcome (cure or death).

### Serial detection of SARS-CoV-2

In 10 experimental group patients treated with electrolyzed saline, nasopharyngeal and oropharyngeal samples were collected with swabs in 2.5 ml of viral transport medium, right before starting treatment and on days 2, 4, 6, and 14, and stored at −80 °C until processing. Viral RNA was isolated utilizing TRIzol (Invitrogen, Carlsbad CA, USA), following the manufacturer’s instructions, and SARS-CoV-2 testing was carried out through the SYBR Green-Based Real-Time RT-PCR, using the previously described methodology [43]. That procedure was not carried out on any of the control group patients.

### Evaluation of hematologic and serologic parameters

In the experimental group, changes in the hematologic parameters were evaluated at baseline, at 48 h (day 2), and on days 4, 6, 9, and 14. Complete blood count was evaluated using Sysmex XP-300 (Roche®, Basel, Switzerland) equipment, the biochemical tests for kidney function and liver function were carried out using Cobas c111 (Roche®, Basel, Switzerland) equipment and the serum concentration of testosterone and cortisol (supplementary material) were determined by immunofluorescence with the iCHROMA (Boditech Med Inc. Korea) equipment. The testoterone-cortisol ratio was calculated by dividing the two hormone levels both expressed in nm/L [44]. Patients with any kind of steroidal or hormonal treatment were excluded. Systemic inflammation markers (erythrocyte sedimentation rate and C-reactive protein) were also evaluated and rapid staining of blood smears with staining kits (Hycel, Mexico, Mexico) were performed to quantify: 1) reactive lymphocytes, also called virocytes, 2) large granular lymphocytes, a representation of natural killer cells or cytotoxic T lymphocytes, 3) activated monocytes, and 4) monocytes with aberrant nuclei (clumped chromatin) and basophilic cytoplasm [45][46][13].

### Blinding

Only the researchers that evaluated treatment effectiveness through the VAS, MCII, and PASS instruments answered by the patients, and those that performed the statistical analyses, were blinded.

### Sample size

The sample size calculation was based on the number of patients that had disease progression (hospitalization or death). Ten percent progression in the experimental group and 35% progression in the control group were calculated. Those figures were based on local data from the Mexican city of Colima, in which 43% of confirmed patients were hospitalized, according to health authority reports [47]. Thirty-two patients from each group were needed to reach the required power (0.8), when the statistical analysis was performed at the level of the one-tailed alpha (0.05). At the end of the study, the statistical power for detecting a difference between two distinct groups was calculated (one-tailed alpha = 0.05), utilizing the number of patients with disease progression, resulting in 80.9%. The study was ended when the number of required sample of patients (plus 20%) was achieved.

### Statistical analysis.

Data were presented as the mean ± standard deviation (for data with normal distribution), median with the 25th and 75th percentiles (for data with non-normal distribution), or percentages. Normal data distribution was first determined using the Kolmogorov-Smirnov test and the equality of variances was confirmed using the Levene’s test. The VAS pain quantification and other numerical data with normal distribution (e.g., body mass index or age) were compared between groups, utilizing the Student’s t test. The categorical values were compared, using the Fisher’s exact test. The Wilcoxon signed-rank test was utilized to compare the numerical data with non-normal distribution between groups. For the oxygen saturation parameter, the change from baseline was used to observe the absolute differences between the evaluation periods, calculated through the value after intervention minus the value at baseline, in each patient, which is an acceptable manner for analyzing trial results with baseline and post-treatment measurements [48]. Intra-group comparisons, before and after the analysis of the two related samples, were made to compare the blood and serum values, using the Student’s t test or Wilcoxon signed-rank test, for data with or without normal distribution, respectively. Binary logistic regression analyses were employed to determine the probability of hospitalization or achieving PASS on day 5 (binomial outcome: yes or no) with the experimental treatment, compared with the usual medical care. Data were summarized as relative risks (RRs) with 95% confidence intervals (CIs) and P-values, adjusted for age group, sex, obesity, diabetes, hypertension, progression time, and baseline severity. Binomial regression is considered the most adequate choice for estimating RRs in multivariate analyses [49–51].

The statistical analysis was carried out using the SPSS version 20 software (IBM Corp., Armonk, NY, USA), with the exception of the number needed to treat (NNT), which was calculated employing MedCalc v17.7.2 software (MedCalc Software bvba, Ostend, Belgium), and sample size and statistical power, which were calculated using the online calculator software by HyLown Consulting LLC (Atlanta, GA, USA) to Compare 2 Proportions: 2-Sample, 1-Sided [52], A P < 0.05 was considered statistically significant. Sample size and statistical power were calculated for a one-tailed test. The rest of the analyses were two-tailed tests.

## Results

**Figure 1:**
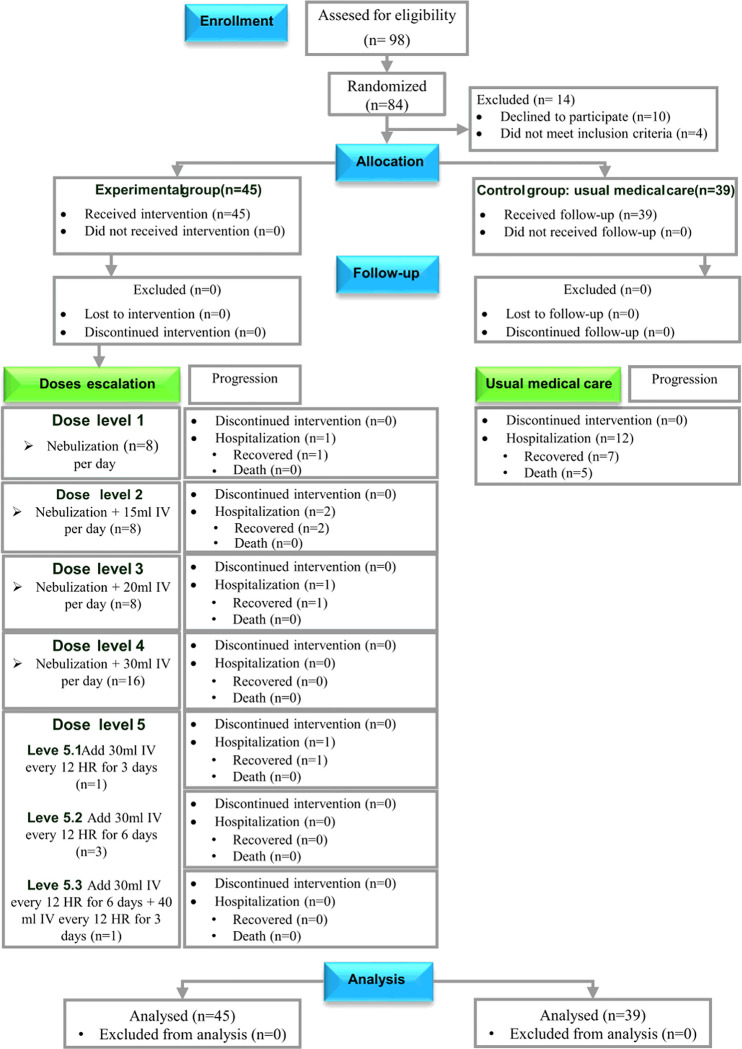
Consort 2010 flow diagram showing the number of patients screened, included, eliminated, and analyzed.

Ninety-eight patients were randomized and screened. A total of 45 patients in the experimental group and 39 patients in the control group agreed to participate in the study, completed it, and were analyzed (Fig. 1). The clinical characteristics of the patients are shown in Table 1.

### Evaluation Of Clinical Improvement And Disease Progression

**Figure 2:**
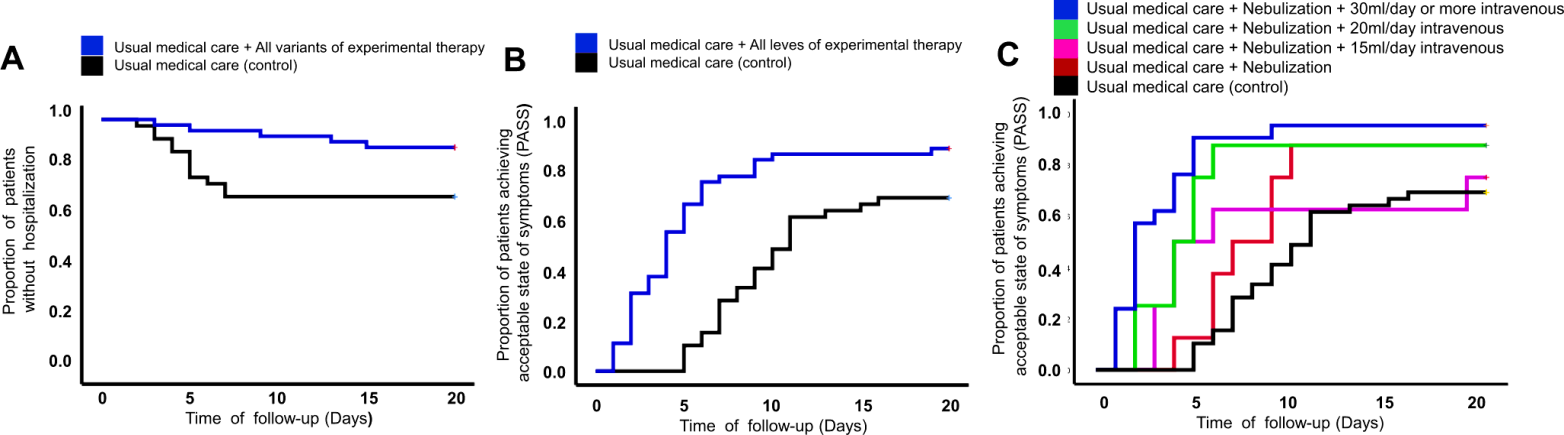
Kaplan-Meier curves showing the progression of patients. The group of patients that received electrolyzed saline had fewer hospitalizations (A) (P=0.020) and achieved an acceptable symptom state (PASS) (B) (P<0.001) in fewer days, compared with the patients that received only usual medical care. Figure C shows that dose level 4+5 was significantly more efficacious for achieving a PASS, with respect to dose level 1 (nebulization) (P=0.007), dose level 2 (15 ml IV application + nebulization) (P=0.033), and usual medical care alone (P<0.001).

The results were analyzed through two data grouping strategies. The control group (usual medical care) was compared with the experimental group, which included all dose levels of the experimental therapy. The other analyses compared the different dose levels of therapy between one another and with the control group, to determine the most efficacious therapeutic dose. In the control group, 30.8% of the patients had disease progression (hospitalization or death), compared with 11.1% of the patients receiving the experimental therapy, with a statistically significant difference in the Kaplan-Meier analysis (P = 0.020) (see Fig. 2A). Regarding only the patients that were hospitalized, the time interval from inclusion in the study to hospitalization was lower in the control group, compared with the experimental therapy group (4.6 ± 1.5 *vs* 9.0 ± 5.1 days, respectively; P = 0.015). Death occurred in 12.8% of the control group patients and 0% of the experimental group patients (P = 0.019). The control group achieved an acceptable symptom state (PASS) on day 11, compared with day 4 in the experimental therapy group (see Table 2). With respect to the different treatment schemes with electrolyzed saline, the dose that included nebulization + intravenous administration of 30 ml/day (dose level 4) or administrations every 12 h (dose level 5) were clearly more efficacious than nebulization alone (dose level 1) or 15 ml/day IV + nebulization (dose level 2) (see Table 2 and Fig. 2B and 2C).

### Progression Of Signs And Symptoms

Table 4 shows improvement in the patient overall self-assessment score in the experimental group at 24 h from the start of treatment (day 1), assessing the progression of the most relevant signs and symptoms of the disease, compared with the control group. Likewise, the experimental group had significant improvement at 24 h in relation to headache, sore throat, retro-orbital eye pain, cough, body temperature, heart rate, and oxygen saturation. Regarding the control group, there was a decrease in fatigue and myalgia on day 3 and significantly reduced arthralgia on day 5. Even the lower dose of the electrolyzed saline, administered only by nebulization, significantly (p < 0.05, for all of the analysis) reduced the sore throat after 24 h of treatment; the myalgia, headache and general condition was improved on day 3 and the fatigue on day 5, with respect to control group.

The means and their standard deviations and the p values obtained through the Student’s t test are shown. Patient overall self-assessment score data, fatigue and pain values on the 0–10 visual analog scale, and heart rate and respiratory rate in units per minute are shown. Oxygen saturation was determined utilizing a pulse oximeter on the right-hand middle finger.

Table 5 shows a symptom analysis with respect to their presence or absence throughout the follow-up. The number of patients with those symptoms at baseline did not differ between groups (except for fatigue, which was higher in the experimental group). The number of patients with myalgia, fever, vomiting, conjunctivitis, and/or anosmia at any time during follow-up was significantly reduced in the experimental group, compared with the control group. There was also a decrease in diarrhea in the experimental group, albeit not statistically significant. With respect to patients with one particular symptom, the last day they presented with fever (1.0 ± 0.4 *vs* 2.6 ± 1.2, P < 0.001), chills (2.2 ± 2.8 *vs* 4.5 ± 4.4, P = 0.041), anosmia (5.3 ± 3.6 *vs* 8.4 ± 4.5 P = 0.021), or ageusia (4.4 ± 3.7 *vs* 9.1 ± 3.9, P < 0.001) was significantly lower in the experimental group *vs* the control group.

The oral administration route was indicated in 5 patients and gargling was indicated in 6, to treat gastrointestinal symptoms and intense sore throat, respectively. With respect to both situations, the patients reported a reduction or disappearance of symptomatology within 24–48 h after administration.

With respect to hospitalized patients, all the experimental therapy group patients recovered, but their follow-up within the hospital was not included in the study protocol. However, there was an exception. One patient requested experimental treatment continuation while hospitalized, which was feasible through the authorization of the ethics committee and the treating physicians. Said patient was 61 years old, morbidly obese, and was intubated the same day of hospital admission, due to pneumonia with multiple foci, lung damage in 90% of the parenchyma, and a sequential organ failure assessment (SOFA) score of 14. During his hospital stay, the patient received azithromycin, hydroxychloroquine, steroids, and two applications of anti-IL-6 antibodies. In 10 days, he was extubated, presenting with multiorgan complications, including kidney failure, for which he required one session of hemodialysis, in addition to four sessions of blood transfusions due to severe anemia. During that period, the patient received electrolyzed saline at a dose of 30 ml every 12 h for the 10 days he was intubated and 20 ml every 12 h for the 6 following days that he was hospitalized and extubated, finalizing treatment with 15 ml per day for 6 days during his at-home recovery. His diagnosis upon hospital discharge was COVID-19 in remission, polyneuropathy of the critically ill patient, anemia, and thrombocytopenia under evaluation. The patient recovered over a three-week period of home care.

### Sars-cov2 Detection During Treatment With Electrolyzed Saline

Serial virus detection in nasopharyngeal and oropharyngeal samples at baseline and on days 2, 4, 6, and 9 was carried out in 10 patients. As shown in Table 6, more than 50% of patients were negative for the virus on day 4, with only a small number of positive patients on day 6. The presence of the virus was negative, in the majority of the cases, in the days after having achieved a PASS. Importantly, patient P30 achieved a PASS on days 3 to 5, but reported an unacceptable state on day 6, and a PASS on day 7 and thereafter. Said case suggests that a PASS does not always accompany the elimination of the virus (positive patient up to day 6) and that there can be symptom relapse. Patient P29 achieved a PASS on day 2, was negative for the virus up to day 6, when she was once again positive. Patients P29 and P30 were a couple, living together, without implementing physical distancing measures during follow-up, signifying that the probable cause of positivity on day 6 of P29 was due to transitory reinfection or contamination derived from living with a patient still presenting with viremia (P30).

### Inflammatory And Immune Response Markers

The erythrocyte sedimentation rate was a parameter that remained elevated during the entire follow-up (see supplementary material), with no significant differences between the baseline value and with the rest of the days evaluated. That was due to the fact that the maximum value reached by each patient varied greatly during the days of follow-up. There was a significant decrease in C-reactive protein (CRP) 48 h after starting treatment, with average reductions of 51% and 71%, at 48 h and 4 days post-treatment, respectively (see supplementary material). Considering baseline CRP values and the patient overall self-assessment score (0–10, very well to very poorly) as 100% and the relative value in the subsequent days of evaluation, there was a significant correlation between CRP and the clinical progression of the patients (r = 0.753, P < 0.001). When there was a greater decrease in CRR, there was a greater reduction in the patient overall self-assessment score (reduced severity).

In relation to the baseline value of hematopoietic cells, there was a significant increase (within normal values) of total leukocytes on days 6, 9, and 14. The quantity of total neutrophils and lymphocytes gradually increased on days 2 and 4, until reaching significantly high levels on day 6. The reactive lymphocytes had no significant changes during the follow-up. The quantity of large granular lymphocytes (a representation of natural killer cells) began to gradually rise, with a median of 77 × 10^3^/μL (25th and 75th percentiles, 47–88) at baseline, until being significantly high on day 6, with 158 × 10^3^/(μL (25th and 75th percentiles, 91–214) (P = 0.028), after which they began to decrease. The quantity of total monocytes gradually decreased, with no significant differences. However, the aberrant monocytes (larger cells, with clumped chromatin and basophilic cytoplasm) decreased significantly, with a median of 430 × 10^3^/μL (25th and 75th percentiles, 126–762) at baseline, to 184 × 10^3^/μL (25th and 75th percentiles, 49–487) in 48 h (P = 0.043). That decrease was sustained during the entire follow-up. The activated monocytes had no significant changes, with respect to baseline values, during the follow-up. Another change was an increase in platelets, which although they remained within normal values, they rose consistently throughout the follow-up, having significantly high values on days 6 to 14 (see supplementary material).

The quantity of total monocytes correlated with CRP levels (r = 0.337, P = 0.024). Most interestingly, the percentage of change in the aberrant monocytes correlated with the percentage of change in the patient overall self-assessment score (r = 0.581, P < 0.001), signifying that the more the aberrant monocytes decreased, the better the patient felt. The gradual and significant increase of platelets after treatment correlated with several beneficial aspects, such as less inflammation, increased lymphocytes, and clinical improvement of the patients, given that the quantity of platelets correlated with CRP values (r= −382, P = 0.028), with total lymphocytes (r = 340, P = 0.002), and with the patient overall self-assessment score (r= −360, P = 0.001).

### Testosterone And Cortisol Levels

The concentration of cortisol significantly decreased on day 2. On the other hand, testosterone concentration increased, although not statistically significant. There was a significant increase of testosterone-cortisol ratio on days 2, and 4. The gradual and significant decrease in cortisol after treatment correlated with increased lymphocytes (r= −0.293, P = 0.017), whereas the increase in testosterone-cortisol ratio correlated with the decrease in activated monocytes (r= −342, P = 0.039).

### Adverse Events And Toxicity

One patient (dose level 2) did not tolerate the nebulizations, due to a burning sensation in the throat, and stopped using them on the second day, but continued with IV applications. Two patients reported transitory dizziness lasting for 10 min, after the intravenous application of the experimental solution. It was self-limited and controlled by lying down. No other adverse events were reported. Eighteen percent of the patients in the experimental group stated they had nightmares at some time during follow-up, but with no statistical difference from the 10% in the control group that also experienced them (P = 0.253). There were no abnormal or unexpected alterations due to COVID-19 in the serum analyses of liver enzymes (ALT, AST, LDH, ALP) bilirubin, albumin, glucose, creatinine, uric acid, urea, or complete blood count test (see supplementary material). It is important to point out that the administration of electrolyzed saline caused no unfavorable interaction with the medications the patients were taking. Nevertheless, that observation was based on the symptomatology reports of the patients and not on biochemical analyses of possible interactions.

## Discussion

In ambulatory COVID-19 patients under usual medical care, the additional administration of electrolyzed saline reduced the probability of disease progression (hospitalization and death) by 90%, compared with ambulatory patients treated with usual medical care alone. Different signs and symptoms, such as body temperature, oxygen saturation, headache, cough, and sore throat, improved significantly after the first 24 h of the experimental therapy and the time for achieving an acceptable symptom state (PASS) was significantly reduced.

Administration by nebulization (dose level 1) of electrolyzed saline induced a beneficial effect, reducing the time for achieving a PASS from 11 days (with usual medical care alone) to 7. Nevertheless, the treatment was most efficacious when high doses of intravenous electrolyzed saline (30 ml or more, per day) were added. In that final treatment scheme (dose levels 4 and 5), a PASS was achieved in 2 days. The beneficial effects of the administration of electrolyzed saline can generally be associated with the mechanisms related to: 1) the reduction of inflammatory processes and 2) the elimination of the virus by the immune system and by direct contact with the electrolyzed saline.

The improvement of signs and symptoms correlated with a significant reduction of systemic inflammation, with a decrease of > 50% of C-reactive protein (CRP) levels at 48 h after starting treatment. There was also a correlation between CRP levels and the quantity of monocytes. Said reduction, particularly of aberrant monocytes, was significant at 48 h and lasted to the end of follow-up, strengthening the hypothesis of the modulating effect the systemic administration of electrolyzed saline has on inflammation, reflected in the clinical improvement of the patients. In the early stage of COVID-19, C-reactive protein levels have previously been shown to reflect the extent of lung lesions and disease severity, providing an important clinical evaluation index [53]. Monocytes and pulmonary monocytes play a key early role in the progression to severe COVID-19, by promoting a cytokine storm, ARDS, and disseminated peripheral tissue damage [13]. The aberrant monocytes that decreased after the experimental treatment were larger than normal monocytes, with clumped chromatin and basophilic cytoplasm [46]. Morphologically altered monocytes, especially larger ones, are associated with a hyperinflammatory gene expression profile and with admission to intensive care units in type 2 diabetes patients with COVID-19 [54]. In contrast, with the reduction in the quantity and relative percentage of aberrant monocytes seen after the experimental treatment, the number of normal monocytes increased. Patients with a high number of normal monocytes have a better outcome, with earlier recovery and discharge from the hospital [55]. That finding has been postulated to be relatively specific for COVID-19, as a similar pattern in patients with other viral illnesses, such as H1N1 influenza, HIV, or hantavirus, has not been seen [55].

In relation to improved immune function, through the administration of electrolyzed saline, a gradual increase in total lymphocytes and large granular lymphocytes (a representation of natural killer cells) was observed, reaching a significantly high level on day 6. Lymphocytes play a crucial role in virus clearance after a viral infection. On the one hand, natural killer (NK) cells eliminate virally infected cells via degranulation, receptor-mediated apoptosis, and antibody-dependent cell-mediated cytotoxicity [56]. On the other hand, the humoral immune response, primarily mediated by the production of antibodies by plasma B cells (B lymphocyte-derived cells), plays a role in the neutralization of the virus [57]. Coinciding with the results of the present work, the lymphocyte count and the number of NK cells have been postulated to correlate with disease severity and may serve as a tool for identifying patients with a more severe clinical presentation of SARS and COVID-19 [56][58]. A lymphocyte count of less than 1.5 × 10^9^/L may be useful in predicting the severity of clinical outcomes [59]. Even though T lymphocytes were not specifically identified in the present study, the large granular lymphocytes observed are a type of T lymphocyte. Previous studies have shown that the time of recovery of the T lymphocyte count was fairly consistent with the clinical course [57]. In an improved subgroup of severe patients, the value of T lymphocytes was reported to begin to increase after 15 days of treatment, finally returning to normal levels after 25 days of treatment. In contrast, the level of T lymphocytes in a subgroup of severely ill patients continued to fall, until their deaths [57]. That behavior concurred with the variations in the number of the large granular lymphocytes found in the present study, in which that special type of lymphocyte increased on day 6 of treatment, in accordance with the clinical improvement of the majority of patients, and began to decrease in quantity on day 9. The speed with which the process of elevation and reduction in those cells took place should be mentioned.

Another relevant aspect was the constant and significant increase in platelets, after treatment with electrolyzed saline. Yang et al. (2020) recently demonstrated an association between reduced platelets and mortality in patients with COVID-19 [60]. Jecko Thachil (2020) correctly interpreted those results [60] as follows: 1) the ‘higher’ platelet counts for an illness as severe as COVID-19 is unusual, and likely points towards liver activation and thrombopoietin release; 2) the lung-specific entry of SARS-CoV-2 suggests that the lung megakaryocytes, in response to liver thrombopoietin, locally produce a large number of platelets to help with the defense of the host; 3) the reduction of platelets in patients with severe disease could be due to the fact that the platelets are being consumed to form pulmonary thrombi, which occurs when multiple efforts (including those of the platelets) to stop the infection have not succeeded, and blocking the viral invasion has become necessary; and 4) Yang et al. also showed that mortality decreased with the increasing of the platelet count, suggesting the thrombotic process has abated and platelets are no longer consumed into the clot [60]. In addition, platelets also have an anti-inflammatory potential by regulating macrophage functions, regulatory T cells, and secreting pro-resolving mediators [61]. All those observations concur with the findings of the present study, in which the increase in platelets correlated with less inflammation (reduced CRP levels), an increase in total lymphocytes, and clinical improvement in the patients (a lower patient overall self-assessment score).

Cortisol and testosterone are hormones related to immune system regulation [62]. The increase in testosterone found in the present study (although not statistically significant) is in agreement with recent findings reporting that low testosterone levels are associated with immune system deficiencies and greater severity of COVID-19 [63]. Likewise, low levels of cortisol, found in the present study, correlated with increased lymphocytes, which can contribute to a better antiviral response by the body. It has recently been found that high cortisol levels are associated with greater risk of death in COVID-19 patients [64]. Similarly, here we report an increase in testosterone-cortisol ratio on days 2 and 4, post-treatment. This is a parameter not previously studied in patients with COVID-19. This increase was correlated with a reduction in activated monocytes, which can help to reduce the systemic inflammatory process. Monocyte activation are abnormal and contribute to the COVID-19 cytokine storm by releasing massive amounts of pro-inflammatory cytokines [65][13].

The influence of testosterone and cortisol on monocytes has been previously reported. In diabetic patients with hypogonadism, testosterone therapy reduced inflammatory activation of human monocytes [66]. It has also been found that cortisol signaling through the mineralocorticoid receptor, under oxidative stress, may promote monocyte inflammatory activation [67][68], so a reduction in cortisol would also be favoring the reduction of activated monocytes, especially in the context of rising testosterone levels. Further, based on the assumption that free testosterone is a marker of anabolism, while cortisol is indicative of catabolism, it has also been found that an increase in the testosterone-cortisol ratio is favorable for protein anabolism [44][69], which could be beneficial in patients with COVID-19.

In addition, it is well known that electrolyzed saline, also known as electrolyzed water, has an important antiseptic effect [70], when used directly on contaminated tissues or fluids [71][72]. Thus, in addition to the immunomodulatory effect produced when administered systemically, it may inactivate the new coronavirus, by degradation of the envelope and nucleocapsid proteins [73][74], when administered locally to the lungs and throat, via nebulization and/or gargling. Nevertheless, the present study is the first to reveal the remarkable immunomodulating effect of electrolyzed saline, when administered systemically at the proper concentration of active species of chlorine and oxygen, acting to control and limit COVID-19 disease. Importantly, all the results presented herein concur with the rapid elimination of the virus from the respiratory tract, occurring within a few days, with negative virus results in 60% and 80% of the patients on days 4 and 6, respectively.

Local administration of electrolyzed saline in the throat to control pain or its oral intake to control the gastrointestinal symptoms of nausea, vomiting, or diarrhea, were successful in reducing or eliminating said symptomatology in 24 to 48 h, which is congruent with previous reports. In fact, the company supplying the product utilized in the present study (*Esteripharma S.A. de C.V*) has presentations for intranasal (*EsteriFlu®*) and buccopharyngeal (*Estericide® Bucofaríngeo*) applications, as antiseptics that inactivate viruses and eliminate bacteria. However, it is likely that electrolyzed saline, besides having a direct effect on the SARS-CoV-2 in the throat, also has an analgesic and regenerative effect on the epithelium at the local level [75]. The oral route for electrolyzed saline has already been shown to have no adverse effects in preclinical trials [76]. Utilized in pigs to treat porcine epidemic diarrhea virus (PEDv) infection, symptom duration in the infected pigs was markedly lessened and symptom severity was also reduced, producing a much higher survival rate [77]. The oral route for aqueous H_2_, a component of electrolyzed saline, has potent local and systemic anti-inflammatory effects, along with regulating effects on the immune system [28], which could be involved in the mechanism for improving gastrointestinal symptoms.

The administration of electrolyzed saline, as has been shown, has positive regulating effects on the immune system in patients with COVID-19, given that its composition is very similar to that of the reactive chlorine species and reactive oxygen species (ROS) produced by the immune system in mammals, which have been described as mediators and modulators of different physiologic processes. Macrophages and neutrophils release ROS to structurally damage invasive pathogens, thus protecting the host against infection [17]. In addition, ROS have recently emerged as a critical second messenger for immune system regulation [17–21] and the control of exacerbated inflammation or tissue repair, in the process of redox signaling [17–21]. There is a consensus on the fact that the maintenance of redox homeostasis is crucial for the appropriate functioning of cellular processes and cell survival.

Evidence of a direct impact of ROS on the life cycles of viruses is very scarce and controversial. Many lines of evidence suggest that marked signs of increased production of ROS accompany all respiratory viral infections, which are associated with cytokine production, inflammation, cell death, and other pathologic processes (58). However, none of the published data are based on direct measurement of ROS levels, but rather on their indirect determination (e.g. quantification of oxidated metabolites) (58). In accordance with the results of the present study, the view that ROS contribute to the suppression of certain respiratory infections through the induction of innate immune responses (58), including T cell receptor signaling and T cell activation, is posited (58).

Examples of mechanisms that sustain the administration of ROS as beneficial in the fight against viral infections are: a) The influenza virus enhances interferon λ1 (IL29) and λ2/3 (IL28A/IL28B) production, via ROS [78]. ROS scavenging or suppression of ROS production leads to the inhibition of IFNλ synthesis and secretion, and in turn, the enhancement of viral replication (58). b) Signal transducers and activators of transcription (STAT) activation has been shown to be a relevant event in the response against different viruses [79]. ROS formation is involved in STAT activation and the subsequent interferon regulatory factor 1 (IRF-1) and IRF-7 gene expression [80]. IRF-1 has been shown to have a role in shaping innate and adaptive antiviral immunity, by inducing the expression of IFN-stimulated genes (ISGs) and mediating signals downstream of IFN-γ [80], contributing to the clinical improvement of patients with viral infection [81].

Antioxidant therapies are also known to ameliorate and improve disease outcome (58). Treatment with molecular antioxidants reduces intracellular levels of influenza virus polymerase, providing a possible mechanism of viral titer reduction, in response to antioxidant treatment. [82] Because electrolyzed saline also contains molecular H_2_, an antioxidant effect is expected [83]. Other antioxidant substances, such as ascorbic acid and vitamin E, have been shown to have the positive effects of decreasing virus replication and inflammation, albeit results in humans have not been conclusive (58).

The role of ROS in the genesis of pathology or the control of viral infections is extensive and controversial. Our results support the view that their administration is beneficial, having an effect that varies according to the dose employed. ROS can be produced by different systems, including the NADPH oxidase system, the mitochondrial electron transport chain, and enzymes, such as xanthine oxidase, superoxide dismutase and myeloperoxidase, which is why they are substances that are present in and recognized by human cells. Their administration in the form of electrolyzed saline (intravenous or nebulized) in patients with COVID-19, clearly helps redox homeostasis, resulting in a therapy that is very beneficial to patients.

The present analysis has several limitations. First of all, the study was not placebo-controlled, and the patients were not blinded. Blood samples were not collected from the control group, preventing the comparison between groups of the progression of the different hematologic and biochemical parameters. There was a correlation between the clinical evaluation and the different laboratory parameters in the experimental group, leading to the supposition that the less favorable clinical conditions in the control group would also be accompanied by equally unfavorable laboratory parameters, but that could not be confirmed. Despite the fact that the initial study design included blinded groups and was placebo-controlled, said structure was not accepted by patients and so the alternative design presented herein was developed. In addition, a higher number of inflammation and coagulation markers should be included in future studies, as well as molecular phenotyping of the blood cell strains. Studies with a larger number of patients, both hospitalized and ambulatory, receiving the most effective dose found in our study, are also needed to confirm our findings.

## Conclusion

In conclusion, the intravenous or nebulized administration of electrolyzed saline importantly reduced the symptomatology and risk for disease progression in ambulatory patients with COVID-19. Its administration was well-tolerated and there were no important adverse effects. The treatment effect was mediated by the reduction of inflammation and the apparently increased antiviral immune response, induced by the active species of oxygen and chlorine from the electrolyzed saline that appeared to mimic the effect of physiologic ROS. Further studies are needed to confirm those results.

## Supplementary Material

Supplement

Supplement

## Figures and Tables

**Figure 3:**
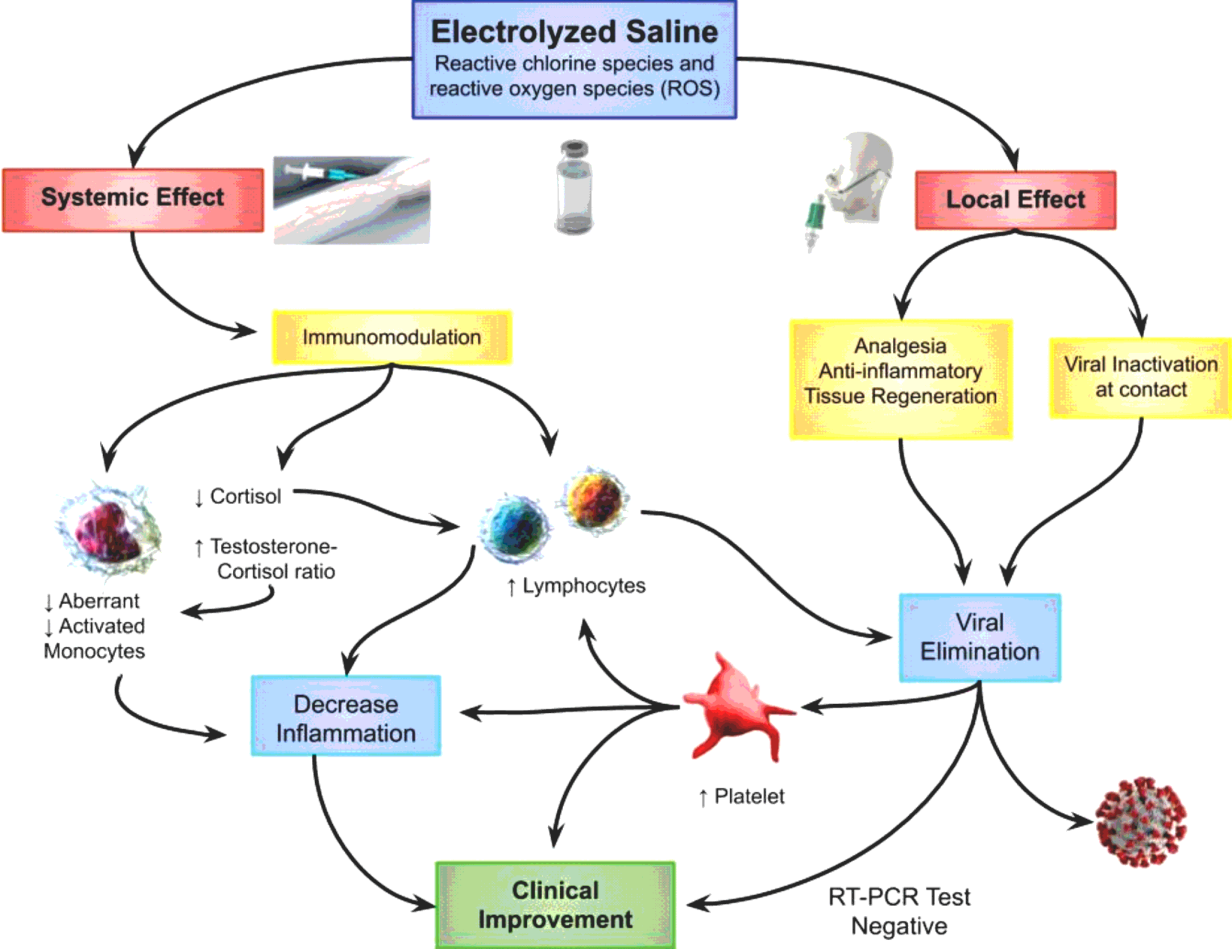
Proposed mechanism of action of the electrolyzed saline in COVID-19 patients. The systemic effect, generated mainly by intravenous application, would generate an immunomodulatory effect that reduces inflammation, with a reduction in aberrant and activated monocytes, as well as an increase in lymphocytes that help eliminate the virus. An increase in platelets and the testosterone-cortisol ratio, with a reduction in cortisol, contribute to this process. A local effect in the airways and digestive tract generates an anti-inflammatory, analgesic and tissue regeneration effect, with the inactivation of the virus by contact. Everything contributes to clinical improvement.

**Table 1:** Main clinical characteristics of the participating subjects at the beginning of the study

Clinical characteristic	Control (n = 39)	Experimental (n = 45)	P
Women (%)	46.2%	46.7%	0.568*
Age (years)	46.0 ± 16.3	48.1 ± 12.9	0.512**
BMI	30.1 ± 4.4	28.9 ± 4.6	0.256**
Diabetes	12.8%	11.1%	0.536*
High blood pressure	17.9%	20.0%	0.517*
Asthma	2.6%	8.9%	0.228
Smoking	2.6%	8.9%	0.228
Progression time	4.1 ± 1.6	4.8 ± 3.4	0.196
SpO_2_	94.4 ± 3.2	93.6 ± 3.6	0.272
Body temperature	37.4 ± 0.9	37.5 ± 0.8	0.696
Patient overall self-assessment	7.0 ± 3.0	6.3 ± 2.3	0.244
Degree of dyspnea	1.4 ± 1.1	1.5 ± 1.3	0.772
**Treatments**	2.4 + 2.0	2.5 + 1.5	0.890
Number Rank	1–7	1–7	
Paracetamol	74.4%	71.1%	0.738
NSAIDs	38.5%	24.4%	0.178
Ivermectin	7.7%	11.1%	0.590
Chloroquine	12.8%	2.2%	0.079
Antibiotics	25.6%	35.6%	0.324
Antivirals	17.9%	31.1%	0.162
Antihistamines	15.4%	15.6%	0.982
Steroids	5.1%	2.2%	0.487
Anticoagulants	17.9%	8.9%	0.235

Percentages or averages and standard deviation are shown. BMI: Body mass index.

* Fisher’s exact test analysis.

** Student’s t test analysis. SpO2: Oxygen saturation: determined by a pulse oximeter on the right-hand middle finger. NSAIDs: nonsteroidal anti-inflammatory drugs; Antivirals: oseltamivir or amantadine; Antibiotics: azithromycin, clarithromycin, or levofloxacin.

**Table 2:** Comparison of time intervals required for achieving an acceptable symptom state in the control and experimentals groups.

Days for achieving a PASS	P* *vs*			
Group (N)	Median	95%CI	Control	Level 4
Control (39)	11.0	9.5–12.5	—	< 0.001
Level 1 (8)	7.0	4.2–9.7	0.078	0.007
Level 2 (8)	4.0	1.2–6.7	0.248	0.033
Level 3 (8)	4.0	1.9–6.0	< 0.001	0.153
Level 4 + 5 (16 + 5)	2.0	1.3–2.6	< 0.001	—
All levels (45)	4.0	3.2–4.8	< 0.001	—

PASS: Patient acceptable symptom state.

* Kaplan-Meier analysis.

The multivariate analysis showed that having received the experimental treatment reduced the risk for becoming hospitalized by 92% (adjusted RR = 0.08, 95% CI: 0.01–0.50, P = 0.007) and resulted in a 43-fold higher probability of achieving an acceptable symptom state on day 5 (adjusted RR = 42.96, 95% CI: 9.22–200.0, P < 0.001) (Table 3). With the experimental treatment, the NNT to prevent one hospitalization was 5.0 (95% CI: 1.41–8.6), signifying that 2 patients needed to be treated with the experimental treatment plus the usual medical care to have one additional patient achieve an acceptable symptom state on day 5 or before (NNT 1.7; 95% CI: 1.35–2.55).

**Table 3:** Binary logistic regression analysis models for hospitalization and achieving an acceptable symptom state.

Covariate	Crude RR* (95% CI)	*P*-*value*	Adjusted** RR* (95% CI)	*P*-*value*
**HOSPITALIZATION**				
Exp. Ther.***	0.28 (0.09–0.89)	0.031	0.08 (0.01–0.50)	0.007
Women	0.18 (0.05–0.70)	0.013	0.09 (0.01–0.63)	0.015
Age group	3.59 (1.91–6.74)	< 0.001	4.12 (1.75–9.67)	0.001
Obesity†	1.34 (0.71–2.53)	0.360	0.93 (0.33–2.59)	0.891
Diabetes	3.12 (0.77–12.68)	0.110	2.71 (0.34–21.53)	0.344
Hypertension	4.51 (1.36–14.89)	0.013	2.50 (0.37–16.58)	0.342
Progression time‡	1.05 (0.87–1.26)	0.590	1.08 (0.77–1.51)	0.629
Baseline severity^	1.12 (0.90–1.38)	0.300	0.80 (0.58–1.10)	0.176
**PATIENT ACCEPTABLE SYMPTOM STATE ACHIEVED ON DAY 5**				
Exp. Ther.***	17.5 (5.24–58.44)	< 0.001	42.96 (9.22–200.0)	< 0.001
Women	1.27 (0.53–3.05)	0.589	1.49 (0.43–5.11)	0.526
Age group¥	0.65 (0.42–1.00)	0.051	0.46 (0.23–0.89)	0.022
Obesity†	1.11 (0.64–1.93)	0.702	2.25 (0.91–5.59)	0.079
Diabetes	0.98 (0.25–3.76)	0.974	2.54 (0.32–20.15)	0.377
Hypertension	0.42 (0.12–1.44)	0.169	0.14 (0.17–1.26)	0.081
Progression time‡	1.04 (0.89–1.22)	0.574	0.99 (0.80–1.22)	0.953
Baseline severity^	0.91 (0.77–1.08)	0.303	1.05 (0.82–1.36)	0.670

* Relative risk (RR) with 95% confidence interval (CI) and P Value, calculated by binary -binomial- logistic regression analyses;

** Adjusted for covariates listed in the table.

*** Exp. ther.: Includes patients at all dose levels of electrolyzed saline.

¥ 18–39, 40–49, 50–59, 60 or more years of age;

† Body mass index 20.0–29.9, 30.0–34.9, 35.0–39.9 and 40.0 kg/m^2^ or more;

‡ Time in days between symptom onset and start of therapy;

^ Baseline patient overall self-assessment, using a 10-point VAS, from ‘very well’ (0) to ‘very poorly’ (10).

**Table 4:** The progression of signs and symptoms over time in the control and experimental group patients.

Clinical characteristic				Clinical characteristic			
	Group		P		Group		P
	Control	Experimental			Control	Experimental	
N = 39 N = 45				N = 39 N = 45			
**Patient overall self- assessment**				**Fatigue**			
Baseline	7.0 ± 3.0	6.3 ± 2.3	0.244	Baseline	5.1 ± 3.7	6.1 ± 2.9	0.412
Day 1	7.4 ± 2.9	5.0 ± 2.6	< 0.001	Day 1	5.8 ± 3.7	5.0 ± 2.8	0.263
Day 3	7.8 ± 2.7	3.8 ± 2.9	< 0.001	Day 3	7.2 ± 3.3	3.8 ± 3.0	< 0.001
Day 5	7.5 ± 3.5	2.7 ± 2.5	< 0.001	Day 5	6.8 ± 3.7	2.8 ± 2.5	< 0.001
Day 7	6.2 ± 3.9	1.9 ± 2.1	< 0.001	Day 7	5.9 ± 4.1	1.8 ± 2.1	< 0.001
Day 9	4.0 ± 4.5	1.3 ± 2.1	0.001	Day 9	5.3 ± 4.5	1.1 ± 2.0	< 0.001
**Headache**				**Retro-orbital eye pain**			
Baseline	6.6 ± 3.4	5.7 ± 3.4	0.273	Baseline	2.8 ± 2.8	2.4 ± 2.9	0.502
Day 1	6.4 ± 3.6	3.3 ± 2.8	< 0.001	Day 1	3.0 ± 2.8	1.6 ± 2.6	0.027
Day 3	6.3 ± 3.4	2.6 ± 2.5	< 0.001	Day 3	2.3 ± 2.5	1.2 ± 2.1	0.038
Day 5	5.6 ± 4.0	1.2 ± 1.8	< 0.001	Day 5	1.1 ± 2.0	0.4 ± 1.3	0.059
Day 7	4.9 ± 3.8	0.5 ± 1.0	< 0.001	Day 7	0.8 ± 2.2	0.3 ± 1.1	0.235
Day 9	2.5 ± 3.1	0.2 ± 0.7	< 0.001	Day 9	0.5 ± 1.8	0.2 ± 1.0	0.465
**Arthralgia**				**Myalgia**			
Baseline	3.8 ± 3.9	4.7 ± 3.3	0.273	Baseline	4.7 ± 3.8	4.7 ± 3.3	0.981
Day 1	3.8 ± 4.0	3.6 ± 3.1	0.783	Day 1	4.9 ± 3.7	3.8 ± 3.0	0.133
Day 3	3.0 ± 3.3	2.3 ± 2.6	0.356	Day 3	5.6 ± 3.2	2.6 ± 2.6	< 0.001
Day 5	2.6 ± 3.5	1.3 ± 1.9	0.044	Day 5	5.0 ± 3.7	1.2 ± 1.9	< 0.001
Day 7	2.4 ± 3.1	0.8 ± 1.6	0.007	Day 7	4.3 ± 3.9	0.9 ± 1.6	< 0.001
Day 9	2.3 ± 3.4	0.5 ± 1.2	0.003	Day 9	3.1 ± 3.5	0.5 ± 1.3	< 0.001
**Sore throat**				**Cough**			
Baseline	3.6 ± 2.6	3.4 ± 3.0	0.727	Baseline	5.9 ± 6.1	6.4 ± 6.4	0.723
Day 1	4.0 ± 2.5	2.5 ± 2.3	0.006	Day 1	7.3 ± 7.0	4.5 ± 4.9	0.038
Day 3	3.4 ± 2.9	1.4 ± 1.6	< 0.001	Day 3	5.4 ± 5.5	2.6 ± 3.7	0.009
Day 5	1.7 ± 2.7	0.7 ± 1.2	0.035	Day 5	2.3 ± 3.2	1.5 ± 3.0	0.321
Day 7	0.9 ± 1.8	0.6 ± 1.1	0.433	Day 7	2.2 ± 4.6	1.2 ± 3.8	0.328
Day 9	0.4 ± 0.8	0.2 ± 0.9	0.556	Day 9	1.5 ± 3.0	0.6 ± 2.4	0.181
**Body temperature °C**				**Heart Rate**			
Baseline	37.4 ± 0.9	37.5 ± 0.8	0.679	Baseline	94.5 ± 11.6	91.1 ± 19.6	0.342
Day 1	37.4 ± 0.9	37.0 ± 0.6	0.008	Day 1	93.4 ± 19.6	86.3 ± 16.8	0.046
Day 3	36.9 ± 1.0	36.8 ± 0.7	0.751	Day 3	88.4 ± 20.3	84.3 ± 14.1	0.332
Day 5	36.6 ± 0.9	36.6 ± 0.3	0.662	Day 5	86.7 ± 12.4	82.0 ± 14.3	0.156
Day 7	36.6 ± 0.9	36.5 ± 0.3	0.577	Day 7	79.7 ± 12.1	77.9 ± 10.3	0.532
Day 9	36.7 ± 0.6	36.5 ± 0.2	0.040	Day 9	84.0 ± 14.1	78.3 ± 9.5	0.049
**Oxygen saturation %**				**Respiratory rate**			
Baseline	94.4 ± 3.2	93.6 ± 3.7	0.272	Baseline	23.9 ± 4.0	23.3 ± 4.2	0.506
Change Day 1	0.46 ± 1.44	0.95 ± 2.23	0.006	Day 1	23.3 ± 5.3	21.7 ± 4.2	0.986
Change Day 3	0.55 ± 2.71	1.56 ± 4.31	< 0.001	Day 3	21.9 ± 6.1	20.9 ± 4.5	0.572
Change Day 5	0.66 ± 2.52	2.24 ± 2.67	0.035	Day 5	19.9 ± 3.7	21.4 ± 4.0	0.280
Change Day 7	1.07 ± 2.51	2.56 ± 2.31	0.433	Day 7	19.0 ± 1.9	20.0 ± 3.2	0.510
Change Day 9	0.93 ± 2.63	2.72 ± 2.72	0.556	CDay 9	19.7 ± 1.8	19.4 ± 1.7	0.181

**Table 5:** Number of patients that presented with the main signs and symptoms of COVID-19.

Symptom	Baseline		P	At any time		P
	Control	Experimental		Control	Experimental	
Headache	87.2%	84.4%	0.721	100%	91.1%	0.056
Fatigue	79.5%	95.6%	0.023	100%	95.6%	0.183
Myalgia	76.9%	82.2%	0.547	100%	86.6%	0.018
Sore throat	76.9%	71.1%	0.546	82.1%	86.7%	0.560
Cough	74.4%	75.6%	0.899	89.7%	80.0%	0.218
Retro-orbital eye pain	64.1%	53.3%	0.318	69.2%	64.4%	0.643
Arthralgia	59.0%	82.2%	0.019	89.7%	86.7%	0.664
Fever	56.4%	42.4%	0.194	76.9%	44.4%	0.002
Chills	53.8%	62.2%	0.290	71.8%	63.4%	0.315
Rhinorrhea	46.5%	56.1%	0.407	46.5%	56.1%	0.407
Nausea	47.4%	48.8%	0.533	65.8%	53.3%	0.177
Conjunctivitis	42.9%	22.2%	0.055	51.3%	22.2%	0.005
Anosmia	43.6%	35.6%	0.299	71.8%	46.7%	0.017
Ageusia	41.0%	40.0%	0.550	71.8%	53.3%	0.065
Dizziness	30.8%	31.1%	0.581	48.7%	46.7%	0.512
Vomiting	20.5%	11.1%	0.188	46.2%	13.3%	0.001
Diarrhea	17.9%	17.8%	0.603	51.3%	35.6%	0.109

**Table 6:** SARS-CoV-2 detection over time in nasopharyngeal samples of 10 patients in the experimental group.

Patient*	Dose level	Age	Baseline severity^	Days with a PASS†	Day of SARS-CoV2 detection				
		(years)			Baseline	2	4	6	9
P1	1	45	3	4	+	+	Neg.	Neg.	Neg.
P12	2	48	8	3	+	+	Neg.	Neg.	Neg.
P18	3	46	9	5	+	+	+	Neg.	Neg.
P19	3	18	2	2	+	+	Neg.	Neg.	Neg.
P21	4	29	5	3	+	Neg.	Neg.	Neg.	Neg.
P22	4	34	10	3	+	+	+	Neg.	Neg.
P29**	4	40	6	2	+	Neg.	Neg.	+	Neg.
P30	5	43	8	7	+	+	+	+	Neg.
P39	4	41	6	1	+	Neg.	Neg.	Neg.	Neg.
P40	5	65	6	2	+	+	+	Neg.	Neg.
	Percent of positivity				100%	70%	40%	20%	0%

* No patient that ended up being hospitalized is shown;

^ Baseline patient overall self-assessment, using a 10-point visual analog scale, from ‘very well’ (0) to ‘very poorly’ (10);

† Days in which the patient achieved an acceptable symptom state (PASS)

** Partner of P30, living together during the entire follow-up. Neg.:Negative

## Abbreviations

ROS: Reactive oxygen species
PASS: Patient acceptable symptom state
RR: Relative Risk
CI: Confidence interval
RPCEC: Cuban Public Registry of Clinical Trials
NSAID’S: Nonsteroidal antiinflamatorie drugs
ARDS: Acute respiratory distress syndrome
HOCI: Hypochlorous acid
H_2_O_2_: Hydrogen peroxide
H_2_: Moleclar hydrogen
RT-PCR: Real time polymerase chain reaction
AST: Apartate aminotransferase
ALT: Alanine aminotransferase
GMP: Good Manufacturing Practices
DLT: Dose-limiting toxicity
RAPID3: Routine Assessment of Patient Index Data 3
VAS: Visual analog scale
RNA: Ribonucleic acid
SOFA: Sequential organ failure assessment
CRP: C-reactive protein
LDH: Lactate dehydrogenase
ALP: Alkaline phosphatase
HIV: Human immunodeficiency virus
NK: Natural killer
PEDv: Porcine epidemic diarrhea virus
IL: Interleucine
NADPH: Nicotinamide Adenine Dinucleotide Phosphate

## References

[1] Cascella M, Rajnik M, Cuomo A, Dulebohn SC, Di Napoli R. Features, Evaluation and Treatment Coronavirus (COVID-19). Treasure Island (FL); 2020. 32150360

[2] Caldera-Villalobos C, Garza-Veloz I, Martínez-Avila N, Delgado-Enciso I, Ortiz-Castro Y, Cabral-Pacheco GA, The Coronavirus Disease (COVID-19) Challenge in Mexico: A Critical and Forced Reflection as Individuals and Society. Front public Heal. 2020;8:337. doi:10.3389/fpubh.2020.00337 PMC7332544 32671012

[3] O'Keefe JB, Tong EJ, Datoo O'Keefe GA, Tong DC. Predictors of disease duration and symptom course of outpatients with acute covid-19: a retrospective cohort study. medRxiv. 2020;:2020.06.05.20123471. doi:10.1101/2020.06.05.20123471

[4] Tenforde MW, Kim SS, Lindsell CJ, Billig Rose E, Shapiro NI, Files DC, Symptom Duration and Risk Factors for Delayed Return to Usual Health Among Outpatients with COVID-19 in a Multistate Health Care Systems Network - United States, March-June 2020. MMWR Morb Mortal Wkly Rep. 2020;69:993–8. doi:10.15585/mmwr.mm6930e1 32730238 PMC7392393

[5] Wang Y, Zhang D, Du G, Du R, Zhao J, Jin Y, Remdesivir in adults with severe COVID-19: a randomised, double-blind, placebo-controlled, multicentre trial. Lancet. 2020;395:1569–78. doi:10.1016/S0140-6736(20)31022-9 32423584 PMC7190303

[6] Baqui P, Bica I, Marra V, Ercole A, van der Schaar M. Ethnic and regional variations in hospital mortality from COVID-19 in Brazil: a cross-sectional observational study. Lancet Glob Heal. 2020;8:e1018–26. doi:10.1016/S2214-109X(20)30285-0 PMC7332269 32622400

[7] Liang L-L, Tseng C-H, Ho HJ, Wu C-Y. Covid-19 mortality is negatively associated with test number and government effectiveness. Sci Rep. 2020;10:12567. doi:10.1038/s41598-020-68862-x 32709854 PMC7381657

[8] Berumen J, Schmulson M, Alegre J, Guerrero G, Olaiz G, Wong-Chew RM, Risk of infection and hospitalization by Covid-19 in Mexico: a case-control study. medRxiv. 2020;:2020.05.24.20104414. doi:10.1101/2020.05.24.20104414

[9] Rakedzon S, Khoury Y, Rozenberg G, Neuberger A. Hydroxychloroquine and Coronavirus Disease 2019: A Systematic Review of a Scientific Failure. Rambam Maimonides Med J. 2020;11. 10.5041/RMMJ.10416 PMC7426548 32792041

[10] Bchetnia M, Girard C, Duchaine C, Laprise C. The outbreak of the novel severe acute respiratory syndrome coronavirus 2 (SARS-CoV-2): A review of the current global status. J Infect Public Health. 2020. 10.1016/j.jiph.2020.07.011 PMC7402212 32778421

[11] Mangalmurti N, Hunter CA. Cytokine Storms: Understanding COVID-19. Immunity. 2020;53:19–25. doi:10.1016/j.immuni.2020.06.017 32610079 PMC7321048

[12] Ortiz-Prado E, Simbaña-Rivera K, Gómez-Barreno L, Rubio-Neira M, Guaman LP, Kyriakidis NC, Clinical, molecular, and epidemiological characterization of the SARS-CoV-2 virus and the Coronavirus Disease 2019 (COVID-19), a comprehensive literature review. Diagn Microbiol Infect Dis. 2020;98:115094. doi:10.1016/j.diagmicrobio.2020.115094 PMC7260568 32623267

[13] Pence BD. Severe COVID-19 and aging: are monocytes the key? GeroScience. 2020;42:1051–61. doi:10.1007/si1357-020-00213-0 32556942 PMC7299454

[14] Huang W, Berube J, McNamara M, Saksena S, Hartman M, Arshad T, Lymphocyte Subset Counts in COVID-19 Patients: A Meta-Analysis. Cytom Part A. 2020;97:772–6. doi:10.1002/cyto.a.24172 PMC7323417 32542842

[15] Henry BM, de Oliveira MHS, Benoit S, Plebani M, Lippi G. Hematologic, biochemical and immune biomarker abnormalities associated with severe illness and mortality in coronavirus disease 2019 (COVID-19): a meta-analysis. Clin Chem Lab Med. 2020;58:1021–8. doi:10.1515/cclm-2020-0369 32286245

[16] Li R, Jia Z, Trush MA. Defining ROS in Biology and Medicine. React Oxyg species (Apex, NC). 2016;1:9–21. doi:10.20455/ros.2016.803 PMC5921829 29707643

[17] Forrester SJ, Kikuchi DS, Hernandes MS, Xu Q, Griendling KK. Reactive Oxygen Species in Metabolic and Inflammatory Signaling. Circ Res. 2018;122:877–902. 29700084 10.1161/CIRCRESAHA.117.311401 PMC5926825

[18] George-Chandy A, Nordström I, Nygren E, Jonsson I-M, Postigo J, Collins LV, Th17 development and autoimmune arthritis in the absence of reactive oxygen species. Eur J Immunol. 2008;38:1118–26. 18383034 10.1002/eji.200737348

[19] Tan H-Y, Wang N, Li S, Hong M, Wang X, Feng Y. The Reactive Oxygen Species in Macrophage Polarization: Reflecting Its Dual Role in Progression and Treatment of Human Diseases. Oxid Med Cell Longev. 2016;2016:2795090. 27143992 10.1155/2016/2795090 PMC4837277

[20] Kuhns DB, Alvord WG, Heller T, Feld JJ, Pike KM, Marciano BE, Residual NADPH oxidase and survival in chronic granulomatous disease. N Engl J Med. 2010;363:2600–10. 21190454 10.1056/NEJMoa1007097 PMC3069846

[21] Schieber M, Chandel NS. ROS function in redox signaling and oxidative stress. Curr Biol. 2014;24:R453–62. 24845678 10.1016/j.cub.2014.03.034 PMC4055301

[22] Calabrese EJ, Giordano JJ, Kozumbo WJ, Leak RK, Bhatia TN. Hormesis mediates dose-sensitive shifts in macrophage activation patterns. Pharmacol Res. 2018;137:236–49. 30326267 10.1016/j.phrs.2018.10.010

[23] Mills EL, O’Neill LA. Reprogramming mitochondrial metabolism in macrophages as an anti-inflammatory signal. Eur J Immunol. 2016;46:13–21. 26643360 10.1002/eji.201445427

[24] Prokopowicz ZM, Arce F, Biedron R, Chiang CL-L, Ciszek M, Katz DR, Hypochlorous Acid: A Natural Adjuvant That Facilitates Antigen Processing, Cross-Priming, and the Induction of Adaptive Immunity. J Immunol. 2010;184:824 LP–835. doi:10.4049/jimmunol.0902606 20018624

[25] Reth M. Hydrogen peroxide as second messenger in lymphocyte activation. Nat Immunol. 2002;3:1129–34. 12447370 10.1038/ni1202-1129

[26] Li Z, Xu X, Leng X, He M, Wang J, Cheng S, Roles of reactive oxygen species in cell signaling pathways and immune responses to viral infections. Arch Virol. 2017;162:603–10. 27848013 10.1007/s00705-016-3130-2

[27] Ge L, Yang M, Yang N-N, Yin X-X, Song W-G. Molecular hydrogen: a preventive and therapeutic medical gas for various diseases. Oncotarget. 2017;8:102653–73. doi:10.18632/oncotarget.21130 29254278 PMC5731988

[28] Lin C-P, Chuang W-C, Lu F-J, Chen C-Y. Anti-oxidant and anti-inflammatory effects of hydrogen-rich water alleviate ethanol-induced fatty liver in mice. World J Gastroenterol. 2017;23:4920–34. 28785146 10.3748/wjg.v23.i27.4920 PMC5526762

[29] Le Tourneau C, Lee JJ, Siu LL. Dose escalation methods in phase i cancer clinical trials. J Natl Cancer Inst. 2009;101:708–20. 19436029 10.1093/jnci/djp079 PMC2684552

[30] American College of Allergy A and I. No Title. A Message to Asthma Sufferers About a Shortage of Albuterol Metered Dose Inhalers. 2020;:1 https://www.newswise.com/articles/a-message-to-asthma-sufferers-about-a-shortage-of-albuterol-metered-dose-inhalers. Accessed 23 Mar 2020.

[31] British Lung Foundation. No Title. What is social shielding and who needs to do this? 2020;:1 https://www.blf.org.uk/support-for-you/coronavirus/what-is-social-shielding. Accessed 23 Mar 2020.

[32] Asthma Society of Ireland. No Title. Coronavirus (COVID-19) Advice. 2020;:1 https://www.asthma.ie/news/coronavirus-covid-19-advice.

[33] British thoracic society. No Title. C0VID-19: information for the respiratory community. 2020;:1 https://www.brit-thoracic.org.uk/about-us/covid-19-information-for-the-respiratory-community/. Accessed 23 Mar 2020.

[34] Cohen DR, Todd S, Gregory WM, Brown JM. Adding a treatment arm to an ongoing clinical trial: a review of methodology and practice. Trials. 2015;16:179. doi:10.1186/s13063-015-0697-y 25897686 PMC4457999

[35] Kvien TK, Heiberg T, Hagen KB. Minimal clinically important improvement/difference (MCII/MCID) and patient acceptable symptom state (PASS): What do these concepts mean? Ann Rheum Dis. 2007;66:iii40–1. 17934093 10.1136/ard.2007.079798 PMC2095292

[36] Fei JZ, Perruccio A V, Ye JY, Gladman DD, Chandran V. The relationship between patient acceptable symptom state and disease activity in patients with psoriatic arthritis. Rheumatology. 2019;59:69–76. doi:10.1093/rheumatology/kez202 31199486

[37] Delgado-Enciso I, Valtierra-Alvarez J, Paz-Garcia J, Preciado-Ramirez J, Soriano-Hernandez AD, Mendoza-Hernandez MA, Patient-reported health outcomes for severe knee osteoarthritis after conservative treatment with an intra-articular cell-free formulation for articular cartilage regeneration combined with usual medical care vs. usual medical care alone: A randomized co. Exp Ther Med. 2019;17:3351–60. 30988711 10.3892/etm.2019.7384 PMC6447772

[38] Skipper CP, Pastick KA, Engen NW, Bangdiwala AS, Abassi M, Lofgren SM, Hydroxychloroquine in Nonhospitalized Adults With Early COVID-19. Ann Intern Med. 2020. doi:10.7326/M20-4207 PMC7384270 32673060

[39] Tubach F, Ravaud P, Baron G, Falissard B, Logeart I, Bellamy N, Evaluation of clinically relevant changes in patient reported outcomes in knee and hip osteoarthritis: The minimal clinically important improvement. Ann Rheum Dis. 2005;64:29–33. 15208174 10.1136/ard.2004.022905 PMC1755212

[40] Englbrecht M, Tarner IH, van der Heijde DM, Manger B, Bombardier C, Muller-Ladner U. Measuring pain and efficacy of pain treatment in inflammatory arthritis: a systematic literature review. J Rheumatol Suppl. 2012;90:3–10. 22942322 10.3899/jrheum.120335

[41] Delgado-Enciso I, Paz-Michel B, Melnikov V, Guzman-Esquivel J, Espinoza-Gomez F, Soriano-Hernandez AD, Smoking and female sex as key risk factors associated with severe arthralgia in acute and chronic phases of chikungunya virus infection. Exp Ther Med. 2018;15:2634–42. 29467856 10.3892/etm.2017.5668 PMC5792796

[42] Reginster JY, Dudler J, Blicharski T, Pavelka K. Pharmaceutical-grade Chondroitin sulfate is as effective as celecoxib and superior to placebo in symptomatic knee osteoarthritis: The ChONdroitin versus CEIecoxib versus Placebo Trial (CONCEPT). Ann Rheum Dis. 2017;76:1537–43. 28533290 10.1136/annrheumdis-2016-210860 PMC5561371

[43] Meza-Robles C, Barajas-Saucedo CE, Tiburcio-Jimenez D, Mokay-Ramírez KA, Melnikov V, Rodriguez-Sanchez IR One-step nested RT-PCR for COVID-19 detection: A flexible, locally developed test for SARS-CoV2 nucleic acid detection. J Infect Dev Ctries. 2020;14 07 SE-Coronavirus Pandemic. doi:10.3855/jidc.12726 32794453

[44] Ferrari D, Lombardi G, Strollo M, Pontillo M, Motta A, Locatelli M. A Possible Antioxidant Role for Vitamin D in Soccer Players: A Retrospective Analysis of Psychophysical Stress Markers in a Professional Team. Int J Environ Res Public Health. 2020;17:3484. doi:10.3390/ijerphl7103484 PMC7277111 32429456

[45] Singh A, Sood N, Narang V, Goyal A. Morphology of COVID-19-affected cells in peripheral blood film. BMJ Case Rep. 2020;13:e236117. doi:10.1136/bcr-2020-236117 PMC10577773 32467125

[46] Lüke F, Orsó E, Kirsten J, Poeck H, Grube M, Wolff D, Coronavirus disease 2019 induces multi-lineage, morphologic changes in peripheral blood cells. Ejhaem. 2020;:10.1002/jha2.44. doi:10.1002/jha2.44 PMC7361732 32838398

[47] El Comentario U de C. No Title. Graves, 200 pacientes hospitalizados con Covid-19; hay 21 intubados. June 30, 2020 Accessed 30 Jun 2020.

[48] Vickers AJ. The use of percentage change from baseline as an outcome in a controlled trial is statistically inefficient: a simulation study. BMC Med Res Methodol. 2001;1:6. doi:10.1186/1471-2288-1-6 11459516 PMC34605

[49] McNutt L-A, Wu C, Xue X, Hafner JP. Estimating the Relative Risk in Cohort Studies and Clinical Trials of Common Outcomes. Am J Epidemiol. 2003;157:940–3. doi:10.1093/aje/kwg074 12746247

[50] Wacholder S. Binomial regression in GLIM: estimating risk ratios and risk differences. Am J Epidemiol. 1986;123:174–84. 3509965 10.1093/oxfordjournals.aje.a114212

[51] Diaz-Quijano FA. A simple method for estimating relative risk using logistic regression. BMC Med Res Methodol. 2012;12:14. doi:10.1186/1471-2288-12-14 22335836 PMC3305608

[52] HyLown Consulting LLC. Power and Sample Size HomeCalculators Compare 2 Proportions: 2-Sample, 1-Sided. Calculator. 2013;:1 http://powerandsamplesize.com/Calculators/Compare-2-Proportions/2-Sample-1-Sided. Accessed 5 May 2020.

[53] Wang L. C-reactive protein levels in the early stage of COVID-19. Médecine Mai Infect. 2020;50:332–4. doi:10.1016/j.medmal.2020.03.007 PMC7146693 32243911

[54] ALZAID F, Julia J-B, Diedisheim M, Potier C, Potier L, Velho G, Monocyte class switch and hyperinflammation characterise severe COVID-19 in type 2 diabetes. medRxiv. 2020;:2020.06.02.20119909. doi:10.1101/2020.06.02.20119909 PMC7461002 32816392

[55] Zhang D, Guo R, Lei L, Liu H, Wang Y, Wang Y, COVID-19 infection induces readily detectable morphological and inflammation-related phenotypic changes in peripheral blood monocytes, the severity of which correlate with patient outcome. 2020. doi:10.1101/2020.03.24.20042655

[56] McKechnie JL, Blish CA. The Innate Immune System: Fighting on the Front Lines or Fanning the Flames of COVID-19? Cell Host Microbe. 2020;27:863–9. doi:10.1016/j.chom.2020.05.009 32464098 PMC7237895

[57] He R, Lu Z, Zhang L, Fan T, Xiong R, Shen X, The clinical course and its correlated immune status in COVID-19 pneumonia. J Clin Virol. 2020;127:104361. doi:10.1016/j.jcv.2020.104361 PMC7152870 32344320

[58] National Research Project for SARS BG. The Involvement of Natural Killer Cells in the Pathogenesis of Severe Acute Respiratory Syndrome. Am J Clin Pathol. 2004;121:507–11. doi:10.1309/WPK7Y2XKNF4CBF3R 15080302 PMC7110090

[59] Zhao Q, Meng M, Kumar R, Wu Y, Huang J, Deng Y, Lymphopenia is associated with severe coronavirus disease 2019 (COVID-19) infections: A systemic review and meta-analysis. Int J Infect Dis. 2020;96:131–5. doi:10.1016/j.ijid.2020.04.086 32376308 PMC7196544

[60] Yang X, Yang Q, Wang Y, Wu Y, Xu J, Yu Y, Thrombocytopenia and its association with mortality in patients with COVID-19. J Thromb Haemost. 2020;18:1469–72. 32302435 10.1111/jth.14848 PMC9906135

[61] Margraf A, Zarbock A. Platelets in Inflammation and Resolution. J Immunol. 2019;203:2357 LP–2367. doi:10.4049/jimmunol.1900899 31636134

[62] Taneja V. Sex Hormones Determine Immune Response. Front Immunol. 2018;9:1931. doi:10.3389/fimmu.2018.01931 30210492 PMC6119719

[63] Pozzilli P, Lenzi A. Commentary: Testosterone, a key hormone in the context of COVID-19 pandemic. Metabolism. 2020;108:154252. doi:10.1016/j.metabol.2020.154252 PMC7185012 32353355

[64] Tan T, Khoo B, Mills EG, Phylactou M, Patel B, Eng PC, Association between high serum total cortisol concentrations and mortality from COVID-19. The lancet. Diabetes & endocrinology. 2020;8:659–60. 32563278 10.1016/S2213-8587(20)30216-3 PMC7302794

[65] Merad M, Martin JC. Pathological inflammation in patients with COVID-19: a key role for monocytes and macrophages. Nat Rev Immunol. 2020;20:355–62. doi:10.1038/s41577-020-0331-4 32376901 PMC7201395

[66] Bateman L, Rao R Jones TH, Kelly DM. SAT-049 Testosterone Therapy Reduces Inflammatory Activation of Human Monocytes in Hypogonadal Type-2 Diabetic Men as a Potential Mechanism to Improve Atherosclerosis. J Endocr Soc. 2020;4 Suppl 1:SAT–049. doi:10.1210/jendso/bvaa046.623

[67] Milush J. Role of Monocyte Oxidative Stress and Mineralocorticoid Receptor Signaling on Cardiovascular Disease and Persistent Inflammation in Antiretroviral-Treated HIV+ Persons. Grantome. 2015;:1 https://grantome.com/grant/NIH/R21-HL128123-01.

[68] Gomez-Sanchez E, Gomez-Sanchez CE. The multifaceted mineralocorticoid receptor. Compr Physiol. 2014;4:965–94. doi:10.1002/cphy.c130044 24944027 PMC4521600

