# Supplementary material for: Patient-Reported Health Outcomes After Treatment of COVID-19 with Nebulized and/or Intravenous Neutral Electrolyzed Saline Combined with Usual Medical Care Versus Usual Medical care alone: A Randomized, Open-Label, Controlled Trial
Source: Res Sq. 2020 Sep 10:rs.3.rs-68403. Preprint. [Version 1] doi: 10.21203/rs.3.rs-68403/v1 (PMC7491578; doi:10.21203/rs.3.rs-68403/v1)
Supplement: Supplement [file Additionalfile1.docx]

Laboratory parameters of the patients in the experimental group during follow-up. An analysis comparing baseline values *vs* days 2, 4, 6, 9, and 14 was performed.

| **PARAMETERS** | **DAY** | **MEDIA** | **STANDARD DEVIATIONS** | **P VALUE *vs* BASELINE** |
| --- | --- | --- | --- | --- |
| **Hemoglobin** | 0 | 14.98 | 2.15 |  |
| **(g/dl)** | 2 | 15.25 | 2.57 | 0.996 |
|  | 4 | 14.59 | 1.15 | 0.661 |
|  | 6 | 14.65 | 1.40 | 0.344 |
|  | 9 | 14.41 | 1.18 | 0.583 |
|  | 14 | 14.46 | 1.71 | 0.590 |
| **White Blood Cell count** | 0 | 6.56 | 2.74 |  |
| **(x10^3^/µL)** | 2 | 6.64 | 2.40 | 0.805 |
|  | 4 | 6.86 | 2.32 | 0.812 |
|  | 6 | 7.06 | 2.07 | 0.002 |
|  | 9 | 8.56 | 2.67 | 0.042 |
|  | 14 | 8.44 | 2.39 | 0.045 |
| **Segmented Neutrophil** | 0 | 46.67 | 19.39 |  |
| **(%)** | 2 | 51.09 | 8.44 | 0.597 |
|  | 4 | 56.84 | 9.83 | 0.013 |
|  | 6 | 51.57 | 17.40 | 0.030 |
|  | 9 | 56.08 | 7.35 | 0.055 |
|  | 14 | 48.49 | 24.87 | 0.094 |
| **Segmented Neutrophil** | 0 | 3.39 | 2.69 |  |
| **(x10^3^/µL)** | 2 | 3.32 | 1.56 | 0.948 |
|  | 4 | 3.97 | 1.90 | 0.318 |
|  | 6 | 3.57 | 1.66 | 0.009 |
|  | 9 | 5.11 | 2.42 | 0.137 |
|  | 14 | 4.67 | 2.95 | 0.108 |
| **Lymphocytes** | 0 | 33.04 | 12.72 |  |
| **(%)** | 2 | 32.80 | 10.84 | 0.582 |
|  | 4 | 30.46 | 9.83 | 0.324 |
|  | 6 | 32.90 | 7.15 | 0.024 |
|  | 9 | 30.80 | 6.44 | 0.133 |
|  | 14 | 27.70 | 11.32 | 0.073 |
| **Lymphocytes** | 0 | 1.91 | 0.91 |  |
| **(x10^3^/µL)** | 2 | 2.14 | 0.97 | 0.876 |
|  | 4 | 2.01 | 0.84 | 0.764 |
|  | 6 | 2.56 | 0.86 | 0.017 |
|  | 9 | 2.58 | 0.68 | 0.425 |
|  | 14 | 2.39 | 0.99 | 0.077 |
| **Reactive lymphocytes or** | 0 | 2.0 | 1.5-3.5 |  |
| **virocytes (%)** | 2 | 3.0 | 3.0-4.5 | 0.158 |
|  | 4 | 1.5 | 1.0-5.7 | 0.893 |
|  | 6 | 1.5 | 0.7-3.0 | 0.131 |
|  | 9 | 2.5 | 0.7-4.0 | 0.223 |
|  | 14 | 2.0 | 0.5-3.5 | 0.414 |
| **Reactive lymphocytes or** | 0 | 146.0 | 64.0-180.0 |  |
| **virocytes (x10^3^/µL)** | 2 | 219.0 | 115.5-377.5 | 0.110 |
|  | 4 | 105.0 | 71.0-201.0 | 0.674 |
|  | 6 | 99.0 | 66.0-173.2 | 0.345 |
|  | 9 | 108.5 | 43.5-271.0 | 0.345 |
|  | 14 | 184.0 | 41.0-318.0 | 0.783 |
| **Large granular lymphocytes, a representation of natural** | 0 | 1.0 | 1.0-2.0 |  |
|  | 2 | 2.0 | 0.0-2.0 | 0.783 |
| **killer (%)** | 4 | 0.0 | 0.0-1.7 | 0.234 |
|  | 6 | 1.5 | 0.7-3.7 | 0.414 |
|  | 9 | 1.0 | 0.0-2.0 | 0.317 |
|  | 14 | 0.5 | 0.0-1.7 | 0.414 |
| **Large granular lymphocytes, a representation of natural** | 0 | 77.0 | 47.0-88.0 |  |
|  | 2 | 79.0 | 0.0-109.0 | 0.575 |
| **killer (x10^3^/µL)** | 4 | 0.0 | 0.0-150.0 | 0.866 |
|  | 6 | 158.0 | 91.0-214.0 | 0.028 |
|  | 9 | 84.0 | 0.0-137.0 | 0.917 |
|  | 14 | 44.5 | 0.0-98.7 | 0.465 |
| **Monocytes** | 0 | 12.94 | 8.77 |  |
| **(%)** | 2 | 11.27 | 3.66 | 0.459 |
|  | 4 | 10.01 | 3.52 | 0.141 |
|  | 6 | 9.64 | 3.57 | 0.275 |
|  | 9 | 11.61 | 2.81 | 0.315 |
|  | 14 | 8.12 | 2.99 | 0.243 |
| **Monocytes** | 0 | 0.82 | 0.49 |  |
| **(x10^3^/µL)** | 2 | 0.76 | 0.38 | 0.818 |
|  | 4 | 0.64 | 0.38 | 0.272 |
|  | 6 | 0.67 | 0.47 | 0.779 |
|  | 9 | 0.98 | 0.22 | 0.655 |
|  | 14 | 0.69 | 0.40 | 0.470 |
| **Aberrant Monocytes** | 0 | 8.0 | 3.5-13.5 |  |
| **(%)** | 2 | 4.0 | 1.5-5.5 | 0.018 |
|  | 4 | 4.5 | 1.5-7.5 | 0.027 |
|  | 6 | 4.5 | 0.7-5.2 | 0.027 |
|  | 9 | 1.5 | 1.0-5.2 | 0.027 |
|  | 14 | 3.0 | 1.5-5.2 | 0.043 |
| **Aberrant Monocytes** | 0 | 430.0 | 126.0-762.0 |  |
| **(x10^3^/µL)** | 2 | 184.0 | 49.5-487.0 | 0.043 |
|  | 4 | 236.0 | 30-319.0 | 0.043 |
|  | 6 | 137.5 | 0.0-391.5 | 0.028 |
|  | 9 | 86.5 | 14.5-319.5 | 0.028 |
|  | 14 | 90.5 | 0.0-296.2 | 0.046 |
| **Activated Monocytes** | 0 | 1 | 0.0-3.5 |  |
| **(%)** | 2 | 1.5 | 0.0-4.2 | 0.672 |
|  | 4 | 0.0 | 0.0-2.0 | 0.066 |
|  | 6 | 0.0 | 0.0-2.0 | 0.593 |
|  | 9 | 0.0 | 0.0-1.5 | 0.581 |
|  | 14 | 1.5 | 0.2-2.0 | 0.785 |
| **Activated Monocytes** | 0 | 58.0 | 0.0-138.0 |  |
| **(x10^3^/µL)** | 2 | 79 | 0.0-254.0 | 0.917 |
|  | 4 | 0.0 | 0.0-103.5 | 0.068 |
|  | 6 | 0.0 | 0.0-112.5 | 0.593 |
|  | 9 | 0.0 | 0.0-90.0 | 0.715 |
|  | 14 | 89.0 | 0.0-153.0 | 0.715 |
| **Platelets** | 0 | 230.68 | 60.26 |  |
| **(x10^3^/µL)** | 2 | 249.49 | 63.71 | 0.316 |
|  | 4 | 271.30 | 83.99 | 0.083 |
|  | 6 | 292.75 | 84.01 | 0.005 |
|  | 9 | 332.87 | 68.12 | 0.001 |
|  | 14 | 305.20 | 60.71 | 0.002 |
| **Hematocrit** | 0 | 43.90 | 5.95 |  |
| **(%)** | 2 | 44.02 | 6.19 | 0.863 |
|  | 4 | 42.61 | 3.34 | 0.612 |
|  | 6 | 43.45 | 4.35 | 0.328 |
|  | 9 | 41.39 | 3.28 | 0.273 |
|  | 14 | 41.91 | 5.37 | 0.553 |
| **Red Blood Cell count** | 0 | 5.00 | 0.74 |  |
| **(x10^6^/µL)** | 2 | 5.08 | 0.78 | 0.932 |
|  | 4 | 4.86 | 0.35 | 0.620 |
|  | 6 | 4.89 | 0.43 | 0.356 |
|  | 9 | 4.88 | 0.34 | 0.468 |
|  | 14 | 4.87 | 0.66 | 0.488 |
| **Mean Corpuscular** | 0 | 29.98 | 1.28 |  |
| **Hemoglobin (pg)** | 2 | 29.96 | 1.14 | 0.597 |
|  | 4 | 30.02 | 1.15 | 0.615 |
|  | 6 | 28.43 | 4.18 | 0.300 |
|  | 9 | 29.54 | 0.67 | 0.217 |
|  | 14 | 29.73 | 1.53 | 0.126 |
| **Mean Corpuscular Volume** | 0 | 88.30 | 5.01 |  |
| **(fL)** | 2 | 86.83 | 4.67 | 0.621 |
|  | 4 | 87.71 | 4.39 | 0.985 |
|  | 6 | 89.33 | 4.53 | 0.298 |
|  | 9 | 85.07 | 4.09 | 0.040 |
|  | 14 | 86.51 | 4.76 | 0.406 |
| **Mean Platelet Volume** | 0 | 10.56 | 1.54 |  |
| **(fL)** | 2 | 10.80 | 0.90 | 0.971 |
|  | 4 | 10.75 | 0.84 | 0.901 |
|  | 6 | 9.97 | 1.47825 | 0.328 |
|  | 9 | 10.24 | 0.87 | 0.075 |
|  | 14 | 10.26 | 1.20 | 0.820 |
| **Erythrocyte Sedimentation** | 0 | 20.00 | 13.02 |  |
| **Rate (mm/h)** | 2 | 17.34 | 13.63 | 0.360 |
|  | 4 | 21.98 | 15.08 | 0.729 |
|  | 6 | 19.90 | 15.88 | 0.895 |
|  | 9 | 18.04 | 17.38 | 0.198 |
|  | 14 | 20.34 | 12.63 | 0.883 |
| **Glucose** | 0 | 112.01 | 52.92 |  |
| **(mg/dL)** | 2 | 108.89 | 35.59 | 0.696 |
|  | 4 | 108.81 | 44.55 | 0.519 |
|  | 6 | 114.29 | 54.24 | 0.687 |
|  | 9 | 90.42 | 6.70 | 0.787 |
|  | 14 | 96.83 | 9.17 | 0.482 |
| **Urea** | 0 | 25.41 | 5.736 |  |
| **(mg/dL)** | 2 | 23.77 | 6.18 | 0.431 |
|  | 4 | 24.19 | 5.30 | 0.722 |
|  | 6 | 25.22 | 5.17 | 0.643 |
|  | 9 | 25.50 | 7.82 | 0.514 |
|  | 14 | 25.96 | 7.77 | 0.586 |
| **Creatinine** | 0 | 0.89 | 0.24 |  |
| **(mg/dL)** | 2 | 0.82 | 0.18 | 0.205 |
|  | 4 | 0.79 | 0.18 | 0.050 |
|  | 6 | 0.80 | 0.23 | 0.046 |
|  | 9 | 0.90 | 0.15 | 0.346 |
|  | 14 | 0.83 | 0.26 | 0.556 |
| **Uric acid** | 0 | 5.51 | 1.39 |  |
| **(mg/dL)** | 2 | 4.88 | 1.37 | 0.072 |
|  | 4 | 5.35 | 1.29 | 0.651 |
|  | 6 | 5.74 | 1.97 | 0.906 |
|  | 9 | 6.23 | 1.26 | 0.042 |
|  | 14 | 6.31 | 1.63 | 0.096 |
| **Gamma glutamyl transferase** | 0 | 56.72 | 63.78 |  |
| **(U/L)** | 2 | 58.51 | 58.55 | 0.925 |
|  | 4 | 57.63 | 58.78 | 0.912 |
|  | 6 | 48.11 | 36.19 | 0.302 |
|  | 9 | 43.35 | 37.58 | 0.147 |
|  | 14 | 42.60 | 30.40 | 0.311 |
| **Aspartate aminotransferase** | 0 | 34.95 | 31.48 |  |
| **(U/L)** | 2 | 26.82 | 8.42 | 0.705 |
|  | 4 | 30.62 | 18.11 | 0.194 |
|  | 6 | 30.85 | 14.67 | 0.423 |
|  | 9 | 24.86 | 8.59 | 0.876 |
|  | 14 | 22.23 | 5.62 | 0.169 |
| **Alanine aminotransferase** | 0 | 41.14 | 44.73 |  |
| **(U/L)** | 2 | 31.64 | 15.26 | 0.952 |
|  | 4 | 34.27 | 20.36 | 0.432 |
|  | 6 | 42.16 | 30.67 | 0.780 |
|  | 9 | 29.69 | 9.23 | 0.703 |
|  | 14 | 27.24 | 13.67 | 0.176 |
| **Alkaline Phosphatase** | 0 | 167.56 | 320.76 |  |
| **(U/L)** | 2 | 82.38 | 31.79 | 0.279 |
|  | 4 | 80.10 | 28.61 | 0.261 |
|  | 6 | 73.99 | 28.02 | 0.170 |
|  | 9 | 68.41 | 27.95 | 0.195 |
|  | 14 | 81.23 | 29.72 | 0.277 |
| **Lactic dehydrogenase** | 0 | 196.96 | 39.32 |  |
| **(U/L)** | 2 | 189.98 | 67.76 | 0.170 |
|  | 4 | 213.62 | 49.40 | 0.181 |
|  | 6 | 218.94 | 58.35 | 0.473 |
|  | 9 | 270.02 | 121.17 | 0.400 |
|  | 14 | 169.26 | 35.38 | 0.119 |
| **Total bilirubin** | 0 | 0.34 | 0.41 |  |
| **(mg/dL)** | 2 | 0.32 | 0.28 | 0.728 |
|  | 4 | 0.30 | 0.15 | 0.624 |
|  | 6 | 0.34 | 0.15 | 0.045 |
|  | 9 | 0.31 | 0.21 | 0.426 |
|  | 14 | 0.22 | 0.09 | 0.936 |
| **Direct bilirubin** | 0 | 0.18 | 0.18 |  |
| **(mg/dL)** | 2 | 0.16 | 0.12 | 0.411 |
|  | 4 | 0.15 | 0.06 | 0.446 |
|  | 6 | 0.18 | 0.07 | 0.062 |
|  | 9 | 0.11 | 0.09 | 0.163 |
|  | 14 | 0.12 | 0.06 | 0.545 |
| **Total Protein** | 0 | 6.89 | 0.76 |  |
| **(g/dL)** | 2 | 6.22 | 0.97 | 0.000 |
|  | 4 | 6.22 | 0.667 | 0.000 |
|  | 6 | 6.57 | 0.77 | 0.061 |
|  | 9 | 6.71 | 0.48 | 0.199 |
|  | 14 | 6.17 | 0.40 | 0.251 |
| **Albumin** | 0 | 7.18 | 0.47 |  |
| **(mg/dL)** | 2 | 7.03 | 0.50 | 0.232 |
|  | 4 | 7.04 | 0.61 | 0.503 |
|  | 6 | 7.29 | 0.83 | 0.751 |
|  | 9 | 7.22 | 0.51 | 0.849 |
|  | 14 | 6.71 | 0.27 | 0.413 |
| **Globulin** | 0 | 20.83 | 7.05 |  |
| **(g/L)** | 2 | 20.94 | 8.41 | 0.127 |
|  | 4 | 20.40 | 3.44 | 0.444 |
|  | 6 | 14.25 | 5.40 | 0.026 |
|  | 9 | 21.75 | 3.76 | 0.598 |
|  | 14 | 21.50 | 0.50 | 0.397 |
| **C-reactive protein** | 0 | 41.5 | 52.9 |  |
| **(mg/dL)** | 2 | 20.2 | 27.2 | 0.018 |
|  | 4 | 12.0 | 14.4 | 0.022 |
|  | 6 | 10.5 | 14.9 | 0.017 |
|  | 9 | 7.0 | 15.5 | 0.011 |
|  | 14 | 7.0 | 15.5 | 0.011 |
| **Testoterone** | 0 | 3.45 | 2.42 |  |
| **(ng/mL)** | 2 | 4.04 | 3.12 | 0.223 |
|  | 4 | 4.09 | 3.11 | 0.311 |
|  | 6 | 4.07 | 2.75 | 0.734 |
|  | 9 | 3.84 | 2.85 | 0.704 |
|  | 14 | 3.76 | 2.89 | 0.603 |
| **Seric Cortisol** | 0 | 9.77 | 2.77 |  |
| **(µg/dL)** | 2 | 8.38 | 2.59 | 0.049 |
|  | 4 | 8.17 | 2.78 | 0.064 |
|  | 6 | 9.71 | 3.69 | 0.630 |
|  | 9 | 8.58 | 2.51 | 0.699 |
|  | 14 | 8.67 | 4.49 | 0.209 |
| **Testosterone-Cortisol ratio** | 0 | 0.046 | 0.032 |  |
| **(T/C) **** | 2 | 0.064 | 0.053 | 0.041 |
|  | 4 | 0.060 | 0.038 | 0.048 |
|  | 6 | 0.052 | 0.039 | 0.692 |
|  | 9 | 0.055 | 0.040 | 0.672 |
|  | 14 | 0.067 | 0.080 | 0.289 |
| ****The T/C ratio was calculated by dividing the two hormone levels both expressed in nm/L** | | | | |

Means and standard deviation are shown, together with a before-and-after comparison made using the Student’s t test, except in the parameters marked with an asterisk (*), in which the medians, with the 25th and 75th percentiles, are shown. In the latter, the comparisons of the different times were made using the Wilcoxon test.

Day 2: 48 hours after starting treatment.
